# Digital Twin-Enabled Waveform Optimization for VHF Radio Communication Systems

**DOI:** 10.3390/s26103060

**Published:** 2026-05-12

**Authors:** Chenzhe Zhong, Bo Liu, Wei Zhu, Binnian Wang, Yifan Tan, Xiangchen Wang

**Affiliations:** 1 College of Information and Communication, National University of Defense Technology, Wuhan 430030, China; zhongzhe20@163.com (C.Z.); wangxiangchen20@nudt.edu.cn (X.W.); 2 Department of Information and Communication Command, Information Support Force Engineering University, Wuhan 430030, China; zhuwei929@hotmail.com

**Keywords:** digital twin, VHF communication, waveform optimization, online learning, contextual multi-armed bandit, virtual exploration, adaptive communication, Software-In-The-Loop

## Abstract

Very High Frequency (VHF) radio communication systems face significant challenges in modern electromagnetic environments, including spectrum congestion, dynamic interference, and varying channel conditions. Existing adaptive approaches rely on static rule-based switching or single-cycle optimization, which cannot accumulate operational experience across decision cycles. This paper proposes a digital twin-enabled online learning framework (DT-MAB) for adaptive waveform selection in tactical VHF communication. The framework employs a contextual multi-armed bandit algorithm (Lin-UCB) that continuously learns the mapping from channel conditions to optimal configurations, with the digital twin serving as a virtual exploration sandbox that screens candidate configurations before physical deployment—preventing link disruptions during exploratory actions. An expanded configuration space of 63 candidates (7 waveforms × 3 MAC protocols × 3 power levels) is constructed, and a hierarchical performance evaluation model combining voice quality, bit error rate, communication delay, and transmission range is developed using the Analytic Hierarchy Process (AHP) as the reward function for online learning. Experimental results across 10 random seeds demonstrate that DT-MAB achieves the lowest mean cumulative regret, reducing regret by 29% relative to MAB without a digital twin and by 16.5% relative to PSO-based optimization on average. Ablation experiments confirm that removing virtual exploration increases performance drop events by 49% (from 250 ± 79 to 373 ± 6), demonstrating that the digital twin is a functionally indispensable component of the online learning architecture.

## 1. Introduction

Very High Frequency (VHF) radio communication (30–300 MHz) remains indispensable for military tactical networks, emergency response, maritime operations, and aviation systems [1,2]. VHF instruments are widely deployed for transmitting both regular and emergency information due to their favorable propagation characteristics and infrastructure independence [3]. However, modern electromagnetic environments pose unprecedented challenges: spectrum congestion from proliferating wireless devices causes severe co-channel interference [4,5], mobile ad-hoc network (MANET) topologies demand continuous parameter adaptation [6], with recent work exploring multi-agent deep reinforcement learning for real-time optimization of tactical radio networks [7], and the growing threat of intelligent jamming attacks requires robust anti-interference capabilities [8,9]. These challenges critically degrade VHF system performance and mission effectiveness.

Traditional VHF systems employ fixed waveform configurations that cannot adapt to dynamic channel conditions [10]. While software-defined radio (SDR) enables reconfigurable waveforms encompassing coding, modulation, and protocols [11,12], practical optimization still faces three notable challenges. First, conventional approaches typically optimize individual parameters in isolation [13,14], although cross-layer designs jointly optimizing Adaptive Modulation and Coding (AMC) with Hybrid Automatic Repeat reQuest (HARQ) have shown promise in cellular systems [15]. Second, rule-based adaptation methods lack intelligence [16]—despite recent progress in deep reinforcement learning (DRL)-based Modulation and Coding Scheme (MCS) selection [17] and contextual bandit-based rate adaptation in dynamic wireless environments [18]. Third, offline optimization cannot respond to real-time changes [19], though online RL has demonstrated hardware-validated gains in Wi-Fi [20] and emerging hybrid frameworks combining DRL with federated learning and convex optimization address heterogeneity and information freshness constraints in distributed networks [21]. While these advances have partially mitigated the above challenges in standardized cellular and broadband systems, their direct application to VHF tactical communications remains limited by the absence of standardized Channel Quality Indicator (CQI) feedback, severe bandwidth constraints (typically 25 kHz), and non-standard protocol stacks. These VHF-specific constraints motivate the development of an intelligent, real-time, multi-parameter optimization framework tailored to tactical VHF systems.

Digital twin technology offers a promising paradigm to address these challenges through bidirectional virtual-physical integration [22,23]. By creating high-fidelity virtual replicas synchronized with physical systems, digital twins enable real-time monitoring, predictive simulation, and closed-loop optimization [24,25]. Recent advances have demonstrated successful digital twin applications in 5 G/6 G networks for resource allocation and network management [26,27], with generative AI emerging as a key enabler for wireless network digital twins through hierarchical message-level and policy-level modeling [28]. Machine learning techniques have been integrated into digital twin networks for anomaly monitoring, resource allocation, and model optimization [29], with multi-agent reinforcement learning recently shown effective for large-scale coverage optimization through digital twin-enabled simulation environments [30]. However, existing communication digital twins primarily focus on cellular systems at higher protocol layers, leaving the specific challenges of VHF waveform optimization—including multi-parameter interdependencies and real-time feedback requirements—largely unaddressed [31,32].

The above gaps reveal a fundamental tension in tactical VHF optimization that has not been resolved by existing approaches. The dynamic and adversarial nature of tactical environments demands continuous adaptation that accumulates operational experience across decision cycles—a capability inherent to online learning but absent from existing rule-based, single-cycle, or offline methods. At the same time, online learning in safety-critical communication links faces a stringent exploration–safety trade-off: arbitrary exploratory actions may cause severe link degradation or even communication loss, which is unacceptable for mission-critical tactical operations. Resolving this tension requires both a learning paradigm that retains cross-cycle knowledge and a deployment safety mechanism that prevents harmful exploratory actions—a combination not addressed by prior digital twin communication work focusing on network layer resource allocation [26,27,30] or static optimization [25].

This paper proposes a digital twin-enabled online learning framework (DT-MAB) for adaptive VHF waveform selection that resolves this tension by integrating three coupled mechanisms: (a) a contextual multi-armed bandit algorithm (Lin-UCB) that transforms waveform selection from single-cycle scoring into cumulative online learning, (b) the digital twin elevated from passive simulation to an active virtual exploration sandbox that pre-evaluates each candidate configuration and rejects potentially harmful choices, and (c) an expanded 63-configuration action space with AHP-weighted multi-objective evaluation capturing cross-layer interdependencies among coding, modulation, MAC protocol, and transmit power. The main contributions are as follows:

(1) A three-layer digital twin architecture with Software-In-The-Loop (SITL) synchronization achieving 30 ms average latency, enabling real-time bidirectional interaction between physical VHF equipment and virtual models.
(2) A DT-MAB online learning framework that integrates Lin-UCB contextual bandits with digital twin virtual exploration, providing both cumulative learning capability and deployment safety guarantees for adaptive waveform selection.
(3) Systematic BER characterization of seven waveform configurations under Rayleigh and Rician fading, including comparative analysis of alternative coding-modulation pairings (Turbo + 16QAM, RS + BPSK), with robustness validation across Doppler frequencies and K-factor uncertainty ranges.
(4) Comprehensive experimental validation across 10 random seeds in a 500-round three-phase scenario demonstrating a 29% mean cumulative regret reduction over the no-DT baseline, and ablation experiments quantifying the digital twin’s irreplaceable role (49% increase in link disruption events when virtual exploration is removed).

## 2. System Architecture and Communication Model

### 2.1. Digital Twin Framework

**Figure 1:**
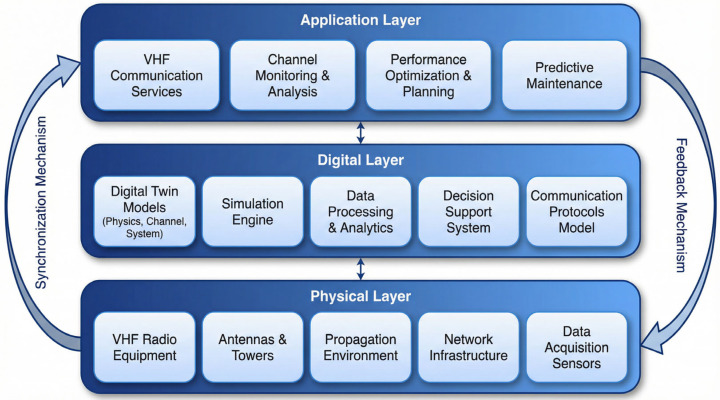
Three-layer digital twin architecture for VHF communication systems.

The proposed digital twin framework adopts a three-layer architecture designed to enable comprehensive modeling, real-time synchronization, and intelligent optimization of VHF communication parameters. Figure 1 illustrates the overall framework structure.

The physical layer encompasses VHF radio equipment, antenna systems, propagation environment, and data acquisition sensors, serving as both data source and optimization target. The digital layer contains virtual replicas coordinated by online learning-based optimization routines (Section 4.6), implemented through MATLAB/Simulink for physical layer simulation and OPNET for protocol simulation. The application layer provides performance monitoring, optimization planning, and predictive maintenance interfaces.

Bidirectional data flow between layers enables two critical mechanisms that distinguish this framework from conventional one-way simulation approaches.

The synchronization mechanism (physical to digital) ensures that digital layer models accurately reflect the real-time state of physical equipment. Sensor data streams are preprocessed, time-stamped, and integrated into the digital model state. Synchronization latency directly impacts the relevance of model predictions and must be minimized.

The feedback mechanism (digital to physical) delivers optimized parameters from the digital layer to physical devices for implementation. Feedback recommendations are validated through simulation before transmission to ensure stability and avoid oscillatory behavior.

To clarify the architectural details depicted in Figure 1, the implementation specifics and data flows of each layer are summarized as follows.

Physical Layer Components. The VHF radio equipment provides software-defined transceiver capabilities supporting reconfigurable coding, modulation, and MAC protocols (Section 3). Antenna systems and the propagation environment together determine the link budget (Equation (3)). Data acquisition sensors continuously capture received signal strength, packet error rate, and MAC collision statistics, sampled at 1 ms granularity and aggregated into the synchronization frame.

Digital Layer Components. The digital twin models include a physics-based channel model (Rayleigh/Rician fading via Jakes’ method, Section 2.2.1), a baseband simulation engine (MATLAB/Simulink, Section 4.1), and a network protocol simulator (OPNET Modeler 18.0, Section 4.1). The data processing module performs SNR estimation, interference level extraction, Doppler estimation, and MAC load computation to construct the context vector $x_{t}$ for the optimizer (Section 4.6.3). The decision support system implements the DT-MAB algorithm (Algorithm 1) including Lin-UCB optimization and virtual exploration. The communication protocols model encodes the MAC state machine and queuing dynamics.
**Algorithm 1** DT-MAB Adaptive Waveform SelectionInput: Exploration parameterα=0.5, safety thresholdε=0.05, digital twin model M Initialize: $A_{k}=I_{4}$, $b_{k}=0$ for all k∈{1,…,63}; $G_{\mathrm{current}}^{*}=0$ For each decision cycle t = 1, 2, … do:Receive context $x_{t}={\left[{\mathrm{SNR}}_{t},I_{t},f_{d,t},\eta_{t}\right]}^{T}$ from digital twin synchronizationFor each arm k = 1, …, 63 do:Compute ${\hat{\theta }}_{k}=A_{k}^{-1}\cdot b_{k}$    Compute ${\mathrm{UCB}}_{k}={\hat{\theta }}_{k}^{T}\cdot x_{t}+\alpha \sqrt{x_{t}^{T}\cdot A_{k}^{-1}\cdot x_{t}}$Select candidate: $k^{*}=\arg\max_{k}{\mathrm{UCB}}_{k}$Virtual exploration via digital twin:    $G_{\mathrm{virtual}}=\mathrm{Simulate}(M,k^{*},x_{t})$ ▷ Evaluate in digital twin    If $G_{\mathrm{virtual}}\geq G_{\mathrm{current}}^{*}-\varepsilon$ then        $k_{\mathrm{deploy}}=k^{*}$ ▷ Accept explorationElse$k_{\mathrm{deploy}}=\arg\max_{k}{\hat{\theta }}_{k}^{T}\cdot x_{t}$ ▷ Revert to safe exploitationDeploy configuration $k_{\mathrm{deploy}}$ to physical VHF linkObserve actual reward $r_{t}$ (AHP score from physical feedback)Update: $A_{k_{\mathrm{deploy}}}\leftarrow A_{k_{\mathrm{deploy}}}+x_{t}x_{t}^{T}$*^*$b_{k_{\mathrm{deploy}}}\leftarrow b_{k_{\mathrm{deploy}}}+r_{t}x_{t}$Update: $G_{\mathrm{current}}^{*}=\max(G_{\mathrm{current}}^{*},\;r_{t})$End For

Application Layer Components. VHF Communication Services route control and data plane traffic to the appropriate physical/digital pathways. Channel Monitoring and Analysis provides operator-facing visualization of link quality. Performance Optimization and Planning interfaces with the optimizer to expose tunable parameters such as the AHP weights (Section 4.5) and the safety threshold ε (Section 4.6.2). Predictive Maintenance leverages historical SNR/error patterns to forecast equipment degradation trends, supporting pre-emptive replacement decisions for tactical units in extended operations.

Inter-Layer Data Flows. Two distinct data flow patterns operate concurrently. The synchronization flow (physical → digital) is a continuous stream of measurement data carried over the SITL interface (Section 4.2) at a 30 ms cycle (validated in Section 4.3), ensuring that the digital twin’s internal state mirrors the physical link in real time. The feedback flow (digital → physical) consists of discrete configuration commands issued at the optimization cycle (1 s per decision), each containing a triplet (waveform index, MAC protocol, power level) that is validated through virtual exploration before deployment. Application-layer interfaces operate at human-readable timescales (5–60 s) for monitoring and configuration adjustment.

### 2.2. Communication System Model

The VHF communication link is modeled as a narrowband fading channel with additive noise. The received signal can be expressed as:

(1) r(t)=h(t)⋅s(t)+n(t)

where $s\left(t\right)$ is the transmitted signal, $h\left(t\right)$ represents the time-varying channel fading coefficient, and $n\left(t\right)$ is additive white Gaussian noise (AWGN) with power spectral density $N_{0}/2$.

#### 2.2.1. Channel Fading Model

The appropriate fading model for VHF links depends on the operational environment and the presence or absence of a dominant line-of-sight (LOS) component. Table 1 summarizes channel model applicability for representative VHF scenarios.

This work targets terrestrial mobile tactical communication in mountainous or valley terrain, where terrain obstruction eliminates reliable LOS paths between ground-mobile nodes. Under these non-line-of-sight (NLOS)-dominant conditions, the Rician K-factor approaches zero and the channel reduces to Rayleigh fading [36]. The fading envelope α=|h(t)| follows a Rayleigh distribution:

(2) $$f(\alpha )=\frac{2\alpha }{\Omega }\exp\left(-\frac{\alpha^{2}}{\Omega }\right),\;\alpha \geq 0$$

where Ω = $E[\alpha^{2}]$ represents the average fading power. The maximum Doppler frequency $f_{d}=v/\lambda$ determines the rate of channel variations, where v is the relative velocity between transmitter and receiver and λ is the carrier wavelength. The applicability of this model to other VHF environments with significant LOS components (e.g., maritime or aeronautical links) would require a Rician model. The framework’s extensibility to such scenarios is validated in Section 3.3.1.

#### 2.2.2. Signal-to-Noise Ratio

The instantaneous signal-to-noise ratio (SNR) at the receiver is:

(3) $$\gamma =\frac{P_{t}\cdot G_{t}\cdot G_{r}\cdot |h{|}^{2}}{N_{0}\cdot B\cdot L}$$

where $P_{t}$ is the transmit power, $G_{t}$ and $G_{r}$ are the transmit and receive antenna gains, B is the signal bandwidth, and L represents path loss. The channel coding and modulation schemes determine how effectively this SNR translates to bit error rate performance.

#### 2.2.3. Modeling Scope and Assumptions

The digital twin operates at the baseband and protocol level: channel coding, modulation, interleaving, multipath fading (Jakes’ model), AWGN, and MAC protocols (Aloha, CSMA, TDMA) are all explicitly simulated in Simulink and OPNET as described in Section 3.2 and Section 4.1. Antenna gain, path loss, and transmit power enter through the link budget equation (Equation (3)) as scalar parameters rather than dynamic models.

RF front-end non-idealities—including power amplifier nonlinearity, local oscillator phase noise, and I/Q imbalance—are not explicitly modeled in the current baseband simulation. Published characterizations of VHF-class equipment [38,39] indicate that these effects introduce a collective implementation loss of 1–3 dB that uniformly affects all waveform configurations without altering their relative BER ranking. This gap is mitigated by the measured SNR feedback path (Section 4.3), which inherently incorporates hardware impairments in the physical layer measurements used for optimization decisions.

Under interference conditions, the effective signal-to-noise-plus-interference ratio at the receiver is:

(4) $$\gamma_{\mathrm{eff}}=\frac{\gamma_{s}}{1+\gamma_{j}}$$

where $\gamma_{s}$ is the signal SNR defined in Equation (3) and $\gamma_{j}=P_{j}\cdot G_{j}/(N_{0}\cdot B\cdot L_{j})$ is the jammer-to-noise ratio with $P_{j}$ denoting jammer transmit power, $G_{j}$ the jammer antenna gain, and $L_{j}$ the jammer-to-receiver path loss. This model is consistent with the narrowband continuous-wave interference configuration used in the experimental validation.

#### 2.2.4. Voice Encoding Model

VHF voice signals are encoded using the Mixed Excitation Linear Prediction (MELP) vocoder at 2.4 kbps, conforming to the MIL-STD-3005 standard for narrowband tactical voice communication. The encoded bitstream is segmented into fixed-length frames and passed to the channel coding module (Section 3.1), where each frame is independently encoded and modulated according to the active waveform configuration. At the receiver, the decoded voice quality depends primarily on the residual bit error rate after channel decoding: BER below 10^−2^ generally produces intelligible speech (VQ ≥ 3 on the 0–5 scale), while BER above 5 × 10^−2^ renders communication unintelligible (VQ ≤ 1). The Voice Quality indicator used in the AHP evaluation (Section 2.3) is derived from this BER-to-intelligibility mapping based on subjective listening tests conducted with the MELP codec under controlled error conditions.

#### 2.2.5. Communication Range Model

The communication range CR is defined as the maximum transmission distance at which the outage probability remains below an acceptable threshold. Under Rayleigh fading, the instantaneous SNR γ follows an exponential distribution and the outage probability—the probability that γ falls below a target threshold $\gamma_{\mathrm{th}}$—is given by:

(5) $$P_{\mathrm{out}}=\mathrm{Pr}(\gamma <\gamma_{\mathrm{th}})=1-\exp\left(-\frac{\gamma_{\mathrm{th}}}{\bar{\gamma }}\right)$$

where $\bar{\gamma }$ is the average received SNR (a function of distance through the path loss L in Equation (3) and $\gamma_{\mathrm{th}}$ is the minimum SNR required to achieve the target BER of 10^−3^ for the active waveform configuration. The communication range CR is determined by solving $P_{\mathrm{out}}$ ≤ ε with ε = 0.1, yielding the maximum distance at which reliable communication is maintained with 90% probability. This distance-dependent metric complements the instantaneous BER indicator by capturing the spatial coverage dimension of communication effectiveness.

It is important to note that the communication range CR defined by Equation (5) is a statistical metric rather than an instantaneous one. The random fading variations of the Rayleigh channel are already integrated through the outage probability formulation and the resulting CR is deterministic for any given combination of waveform configuration and channel statistics. Consequently, CR is sensitive to optimization decisions—particularly the transmit power $P_{t}$ (Equation (3)) and the SNR threshold $\gamma_{th}$ (which depends on the waveform’s BER characteristic, Section 3.3)—and to long-term channel parameters such as the Rician K-factor and Doppler spread, but does not fluctuate with instantaneous fading. This statistical property is precisely what makes CR a stable and meaningful optimization target for the digital twin’s waveform selection decisions, complementing the instantaneous BER metric.

#### 2.2.6. MAC Protocol Model

The single-link SNR expression in Equation (3) characterizes the physical-layer channel quality experienced by an isolated transmission, encompassing path loss, fading, and thermal noise. However, when multiple nodes share the same time-frequency resources in a VHF tactical network, simultaneous transmissions by neighboring nodes produce mutual collisions that effectively act as in-band interference at the receiver, distinct from the external jamming captured by Equation (4). The MAC protocol coordinates channel access among users to manage this network-internal collision behavior. The choice of MAC therefore directly affects communication delay (CD) and effective throughput, complementing the physical-layer SNR optimization addressed by waveform selection.

The data link layer supports three MAC protocols with distinct access mechanisms:

Pure Aloha allows uncoordinated transmission with a theoretical maximum throughput of 1/(2e) ≈ 18.4%. CSMA (Carrier Sense Multiple Access) reduces collision probability by sensing channel occupancy before transmission, achieving higher throughput under moderate traffic loads. TDMA (Time Division Multiple Access) eliminates collisions through pre-assigned time slots but requires synchronization overhead and reduces flexibility under variable traffic patterns.

The MAC protocol selection affects two performance indicators directly: Communication Delay (CD) through queuing and contention-related waiting times, and effective throughput, which determines the packet delivery rate observed at the receiver. These MAC-layer effects are captured by the OPNET protocol simulation (Section 4.1) and reflected in the AHP performance score G through the delay and packet loss components. The interaction between waveform selection and MAC performance—where lower-rate coding increases per-packet airtime and exacerbates MAC-layer congestion—is one of the cross-layer interdependencies that motivates joint optimization across the physical and data link layers.

#### 2.2.7. Digital Twin Synchronization Model

The digital twin maintains an internal state vector $s_{d}(t)$ that mirrors the physical system. The state is updated at each synchronization cycle according to:

(6) $$s_{d}(t+\Delta t)=f\left(s_{d}(t),\;u(t),\;y_{p}(t)\right)$$

where u(t) is the current configuration parameter vector (waveform index, MAC protocol, and power level) deployed on the physical link, $y_{p}(t)$ is the physical layer measurement vector, and Δt = 30 ms is the synchronization period (validated in Section 4.3).

The measurement vector $y_{p}(t)={\left[{\mathrm{SNR}}_{\mathrm{meas}},\;{\mathrm{PER}}_{\mathrm{meas}},\;N_{\mathrm{coll}}\right]}^{T}$ comprises three components obtained through the SITL interface (Section 4.2): ${\mathrm{SNR}}_{\mathrm{meas}}$ is the received signal-to-noise ratio estimated from pilot symbols, ${\mathrm{PER}}_{\mathrm{meas}}$ is the measured packet error rate computed over a sliding window of 50 packets, and $N_{\mathrm{coll}}$ is the MAC collision count within the current synchronization interval. Upon receiving $y_{p}(t)$, the digital twin updates its channel fading model parameters (estimated Doppler spread and average fading power), interference level estimate, and MAC state machine to align the virtual model with the current physical system state. The context vector $x_{t}$ used by the Lin-UCB optimizer (Section 4.6) is then extracted from the updated digital twin state through the signal processing operations described in Section 4.6.3.

### 2.3. Performance Evaluation Metrics

Communication effectiveness is evaluated through four key indicators:

- Voice Quality (VQ): Subjective speech intelligibility score (0–5 scale)
- Bit Error Rate (BER): Ratio of erroneous bits to total transmitted bits
- Communication Delay (CD): End-to-end latency including processing and propagation
- Communication Range (CR): Maximum distance for reliable communication

The overall performance metric combines these indicators through weighted aggregation:

(7) $$G=w_{\mathrm{VQ}}\cdot {\mathrm{VQ}}_{\mathrm{norm}}+w_{\mathrm{BER}}\cdot {\mathrm{BER}}_{\mathrm{norm}}+w_{\mathrm{CD}}\cdot {\mathrm{CD}}_{\mathrm{norm}}+w_{\mathrm{CR}}\cdot {\mathrm{CR}}_{\mathrm{norm}}$$

The weights $w_{i}$ are determined through the Analytic Hierarchy Process (AHP) based on pairwise comparison of metric importance by domain experts, yielding ($w_{\mathrm{VQ}}$, $w_{\mathrm{BER}}$, $w_{\mathrm{CD}}$, $w_{\mathrm{CR}}$) = (0.379, 0.248, 0.253, 0.120) with consistency ratio CR = 0.027 (well below Saaty’s 0.1 threshold). Section 4.5 details the AHP methodology, weight derivation procedure, and quantitative robustness analysis demonstrating that the top configuration ranking is invariant under ±20% perturbation of the baseline weights.

#### Indicator Normalization

Before applying the weighted aggregation in Equation (7), all indicators are normalized to the [0, 1] range using min-max normalization with direction adjustment.

For benefit indicators (higher is better), such as Voice Quality and Range:

(8) $${\mathrm{VQ}}_{\mathrm{norm}}=\frac{\mathrm{VQ}-{\mathrm{VQ}}_{\min}}{{\mathrm{VQ}}_{\max}-{\mathrm{VQ}}_{\min}},\;{\mathrm{CR}}_{\mathrm{norm}}=\frac{\mathrm{CR}-{\mathrm{CR}}_{\min}}{{\mathrm{CR}}_{\max}-{\mathrm{CR}}_{\min}}$$

For cost indicators (lower is better), such as BER and Delay:

(9) $${\mathrm{BER}}_{\mathrm{norm}}=\frac{{\mathrm{BER}}_{\max}-\mathrm{BER}}{{\mathrm{BER}}_{\max}-{\mathrm{BER}}_{\min}},\;{\mathrm{CD}}_{\mathrm{norm}}=\frac{{\mathrm{CD}}_{\max}-\mathrm{CD}}{{\mathrm{CD}}_{\max}-{\mathrm{CD}}_{\min}}$$

The normalization bounds are established based on system specifications and operational requirements: Voice Quality ranges from 0 (unintelligible) to 5 (excellent); BER ranges from 10^−6^ (excellent) to 10^−1^ (poor); Delay ranges from 10 ms (excellent) to 500 ms (unacceptable); Range spans from 1 km (minimum operational) to 50 km (maximum achievable).

For BER, logarithmic transformation is applied before normalization to handle the wide dynamic range:

(10) $${\mathrm{BER}}_{\log}=-\log_{10}(\mathrm{BER}),\;{\mathrm{BER}}_{\mathrm{norm}}=\frac{{\mathrm{BER}}_{\log}-1}{5}$$

This transforms the BER range [10^−6^, 10^−1^] to $B_{log}$ range [1, 6], where $B_{log}$ = 1 corresponds to BER = 10^−1^ (worst) and $B_{log}$ = 6 corresponds to BER = 10^−6^ (best). After normalization, lower BER (better performance) yields higher normalized scores.

## 3. Waveform Design and Performance Analysis

### 3.1. Waveform Configuration Design

In software-defined radio systems, a waveform encompasses the complete signal processing chain rather than merely the physical signal shape, including channel coding, interleaving, modulation, and associated protocol parameters. Rather than designing novel coding or modulation schemes—which is not the focus of this work—we construct a representative waveform configuration set that systematically spans the key design trade-offs relevant to tactical VHF communication. The research contribution lies in the digital twin framework (Section 4) that adaptively selects among these configurations in response to real-time channel conditions, not in the individual waveform designs themselves. The interdependencies among waveform components mean that optimizing them collectively yields greater performance gains than addressing each in isolation.

The five core waveforms are designed to span key trade-off dimensions in tactical VHF communication.

Channel coding spans three levels of error protection. BCH(31,16) and Golay(24,12) offer algebraic decoding with bounded complexity [40], suitable for resource-constrained VHF equipment. RS(31,15) and RS(15,7) operate at the symbol level over GF(2^5^) and GF(2^4^), respectively, providing burst error correction critical under deep fading [41]. Turbo coding (rate 1/3) with 12 MAP decoding iterations approaches Shannon-limit performance at the cost of higher computational latency [42], quantified later in Section 4.1.

Modulation selection covers the coherent/non-coherent boundary. BPSK provides optimal power efficiency through coherent detection (1 bit/symbol). 32-FSK enables non-coherent reception without carrier phase tracking—a decisive advantage in high-mobility scenarios where Doppler-induced phase rotation degrades coherent schemes. 16-QAM (4 bits/symbol) maximizes spectral efficiency but demands accurate phase and amplitude estimation.

Interleaving is matched to the decoder architecture: matrix interleaving for algebraic block decoders (VHF_1 through VHF_4) and pseudo-random interleaving for the iterative Turbo decoder (VHF_5), where statistical independence between decoder input bits is essential for convergence.

Importantly, waveform selection produces cascading effects beyond the physical layer: higher-order modulation reduces per-packet transmission time and thus queuing delay, while low-rate coding increases packet duration and may exacerbate MAC-layer congestion. These cross-layer interactions motivate evaluating waveform configurations through multi-dimensional metrics (Section 4.5) rather than BER alone.

Table 2 summarizes the waveform configurations.

Two additional configurations are included as comparative baselines to address alternative coding-modulation pairings that might appear viable but are suboptimal for VHF tactical conditions:

VHF_6 (Turbo + 16QAM) combines the strongest error correction code with the highest-order modulation in the configuration set. While this pairing maximizes spectral efficiency in theory, 16QAM requires accurate amplitude and phase estimation at the receiver. Under Rayleigh fading, the channel estimation errors that persist during Turbo code convergence (typically in the 3–8 dB SNR region) introduce amplitude distortion that degrades QAM constellation decisions. As a result, VHF_6 exhibits higher BER than VHF_5 (Turbo + BPSK) across the tactical operating SNR range.

VHF_7 (RS(31,15) + BPSK) replaces the 32-FSK modulation of VHF_2 with BPSK while retaining the same RS code. This pairing introduces a structural mismatch: RS(31,15) operates at the symbol level over GF(2^5^), where each symbol comprises 5 bits. In VHF_2, the 32-FSK modulation maps each 5-bit RS symbol directly to one of 32 orthogonal tones, preserving natural symbol-level alignment. With BPSK (1 bit/symbol), each RS symbol must be serialized into 5 consecutive BPSK transmissions, destroying the symbol-level error correction boundary and requiring additional bit-to-symbol demapping at the receiver. This inefficiency is confirmed by simulation, where VHF_7 shows significantly worse BER than VHF_2 across all SNR values.

These two configurations are not included in the core design set but are retained as candidates within the expanded 63-configuration action space (Section 4.6.1), where the DT-MAB algorithm can select them if environmental conditions favor their characteristics. Table 2 is updated to include VHF_6 and VHF_7 as comparative configurations.

To further extend the optimization space beyond waveform and MAC protocol selection, three transmit power levels (5 W, 10 W, and 20 W) are introduced as an additional optimization dimension. The power level affects the received SNR through the link budget equation (Equation (3)) and consequently alters the BER-dependent performance metrics. The resulting configuration space comprises 7 waveforms × 3 MAC protocols × 3 power levels = 63 candidate configurations, providing a sufficiently large action space to validate the online learning approach proposed in Section 4.6.

### 3.2. Simulation Environment

#### 3.2.1. Simulink Implementation

**Figure 2:**
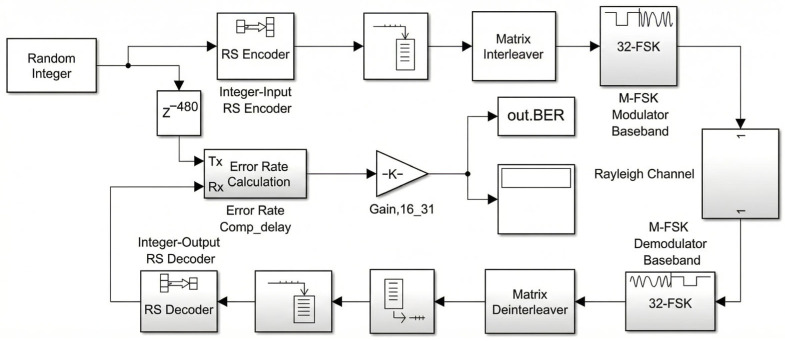
Simulink simulation chain for VHF_2 waveform.

The waveform performance characterization employs MATLAB/Simulink to construct accurate baseband equivalent models. Figure 2 illustrates the simulation chain for VHF_2 (RS + 32FSK) as a representative example.

#### 3.2.2. Channel Model Implementation

The channel model comprises two cascaded components. The Rayleigh fading block implements Jakes’ model with configurable maximum Doppler frequency. For the baseline characterization, $f_{d}=$ 10 Hz corresponds to moderate vehicular mobility (approximately 20 m/s or 72 km/h at 150 MHz VHF carrier frequency, calculated as $v=f_{d}\cdot c/f_{c}$. The AWGN block adds thermal noise with variance determined by the specified $E_{b}/N_{0}$ ratio.

#### 3.2.3. Statistical Methodology

Each BER data point represents the average of at least $10^{6}$ transmitted bits to ensure statistical significance. For low BER regions (<$10^{-4}$), the simulation continues until at least 100 errors are accumulated to maintain confidence interval bounds within acceptable limits. SNR is varied from −5 dB to 15 dB in 1 dB increments to capture the full performance transition region.

### 3.3. BER Performance Results

**Figure 3:**
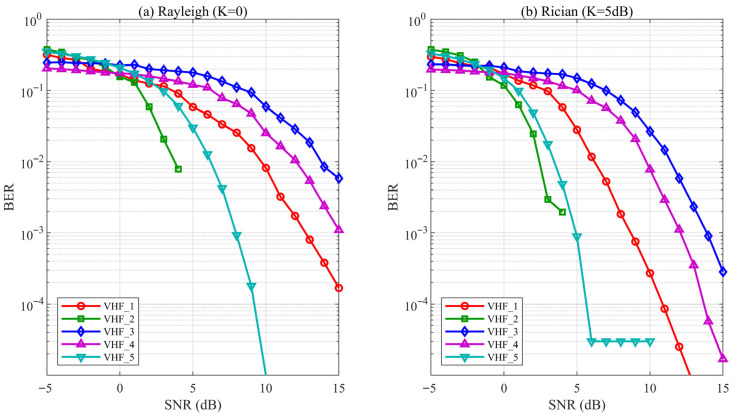
BER performance comparison of five core VHF waveforms: (**a**) Rayleigh fading (K = 0); (**b**) Rician fading (K = 5 dB).

Figure 3 presents the BER versus SNR performance curves for five core waveforms under (a) Rayleigh fading and (b) Rician fading (K = 5 dB) conditions. The figure focuses on the BER transition region (−5 to 15 dB SNR) where waveform selection decisions are most critical. Table 3 provides precise BER values at representative SNR points for cross-reference with the discussion below. Theoretical bound validation is later presented in Table 4. Comparative configurations VHF_6 and VHF_7 are analyzed separately in Section 3.3.2.

Table 3 and Figure 3a reveal a 10 dB spread in required SNR for BER = 10^−2^, ranging from ~4 dB (VHF_2, RS + 32FSK cliff effect) to ~14 dB (VHF_3, Golay + BFSK gradual convergence). VHF_2 and VHF_5 (Turbo + BPSK) reach the simulation floor (<10^−6^) by 5 dB and 15 dB respectively, while the remaining waveforms exhibit gradual BER improvement. This performance spread quantifies the optimization potential available to the adaptive selection framework: Under high SNR conditions, waveform selection can prioritize secondary factors such as latency or spectral efficiency, whereas under low-to-moderate SNR (0–10 dB) the choice has a decisive impact on link reliability.

#### 3.3.1. Channel Model Sensitivity

Figure 3b shows BER curves under Rician fading (K = 5 dB), where the relative waveform ranking is preserved despite a 2–4 dB SNR shift. This confirms that the digital twin’s selection logic extends to Rician scenarios through BER database regeneration without algorithmic modification.

#### 3.3.2. Comparative Waveform Analysis

To validate the design rationale of the core waveform set (Table 2), the BER performance of the two comparative configurations—VHF_6 (Turbo + 16QAM) and VHF_7 (RS + BPSK)—is characterized under Rayleigh fading using the same simulation methodology described in Section 3.2.

**Figure 4:**
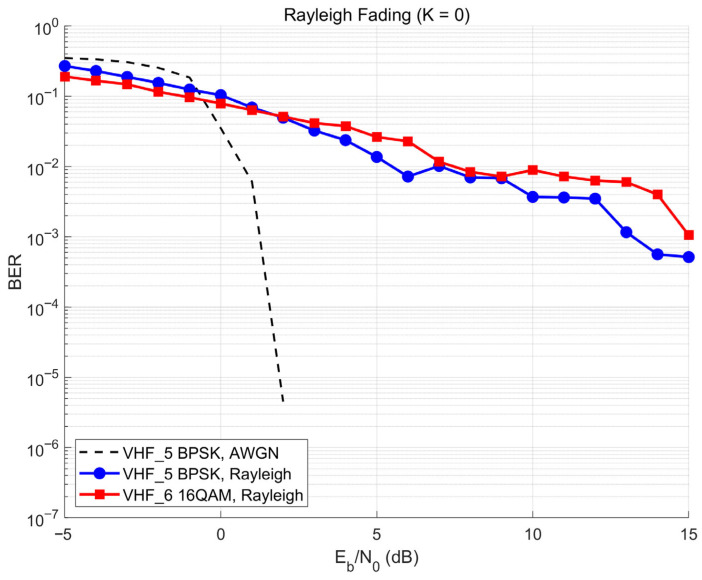
VHF_5 vs. VHF_6 BER comparison.

VHF_6 versus VHF_5. Figure 4 compares VHF_5 (Turbo + BPSK) and VHF_6 (Turbo + 16QAM) under Rayleigh fading, with the AWGN reference for VHF_5 included to illustrate the fading penalty. VHF_6 shows consistently higher BER than VHF_5 across the 5–15 dB operating range (e.g., ~10^−2^ vs. 3.5 × 10^−3^ at 10 dB), because 16QAM’s multi-level amplitude signaling is more sensitive to the amplitude fluctuations of Rayleigh fading; the higher raw symbol error rate overwhelms the Turbo decoder’s correction capability in the medium-SNR regime. This confirms Turbo + BPSK (VHF_5) as the preferred Turbo-coded configuration for the target environment.

**Figure 5:**
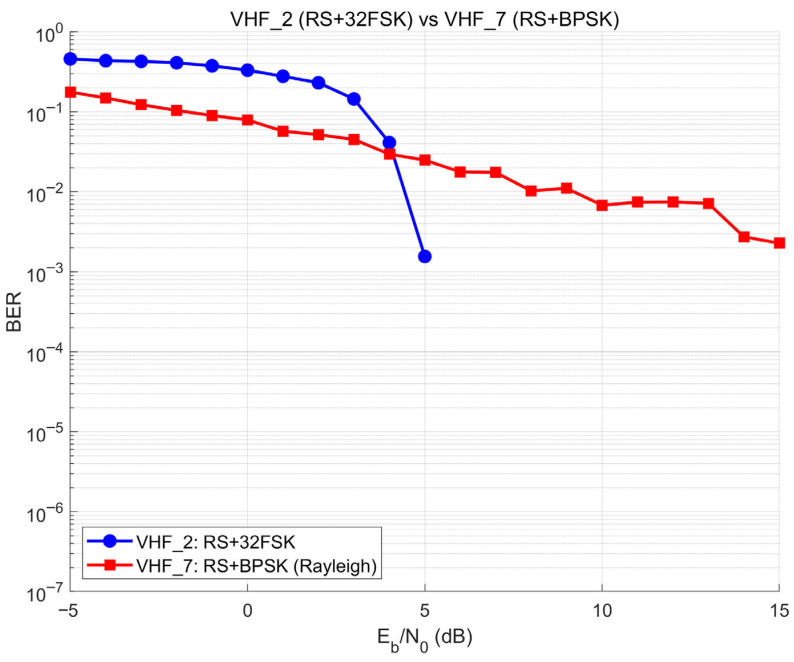
VHF_2 vs. VHF_7 BER comparison.

VHF_7 versus VHF_2. Figure 5 compares VHF_2 (RS + 32FSK) and VHF_7 (RS + BPSK). VHF_2 exhibits the characteristic RS cliff effect (BER drops from 0.15 at 3 dB to 10^−3^ by 5 dB), while VHF_7 shows a gradual decline (~3 × 10^−3^ at 15 dB). The disparity stems from symbol-modulation alignment (as discussed in Section 3.1). In VHF_2, each RS symbol over GF(2^5^) maps directly to one 32-FSK tone, preserving symbol-level error correction. In VHF_7, BPSK serialization breaks this alignment and allows burst errors to span symbol boundaries. Non-coherent FSK detection further provides inherent robustness against carrier phase variations under high-mobility fading. These results validate RS + 32FSK over RS + BPSK for the core configuration set.

#### 3.3.3. Channel Model Robustness Analysis

The BER characterization in the preceding subsections of Section 3.3 uses a fixed Doppler frequency $f_{d}$ = 10 Hz and Rayleigh fading (K = 0). To assess the sensitivity of waveform performance rankings to these channel model parameters, two robustness analyses are conducted.

**Figure 6:**
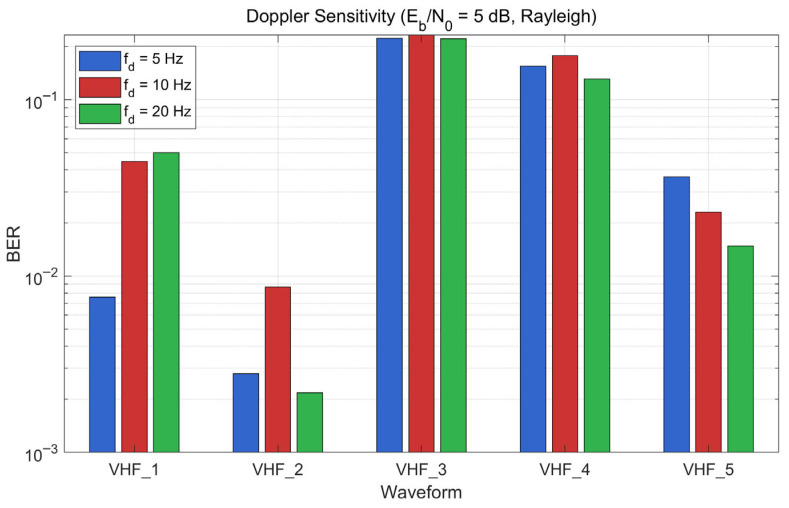
Doppler sensitivity.

Doppler Frequency Sensitivity. Figure 6 presents the BER of five core waveforms at $E_{b}/N_{0}$ = 5 dB under Rayleigh fading for three Doppler frequencies: $f_{d}$ = 5 Hz (pedestrian, ~3.6 km/h), $f_{d}$ = 10 Hz (slow vehicular, ~7.2 km/h), and $f_{d}$ = 20 Hz (fast vehicular, ~14.4 km/h at 150 MHz carrier). The waveform performance ranking remains consistent across all three mobility conditions: VHF_2 achieves the lowest BER (approximately 10^−3^), followed by VHF_1, VHF_5, VHF_4, and VHF_3 (approximately 0.3). While absolute BER values vary by a factor of up to 3–5 between the lowest and highest Doppler settings for individual waveforms, the relative ordering is preserved. This confirms that the waveform selection decisions derived from the baseline $f_{d}$ = 10 Hz characterization remain valid across the range of tactical mobility scenarios.

**Figure 7:**
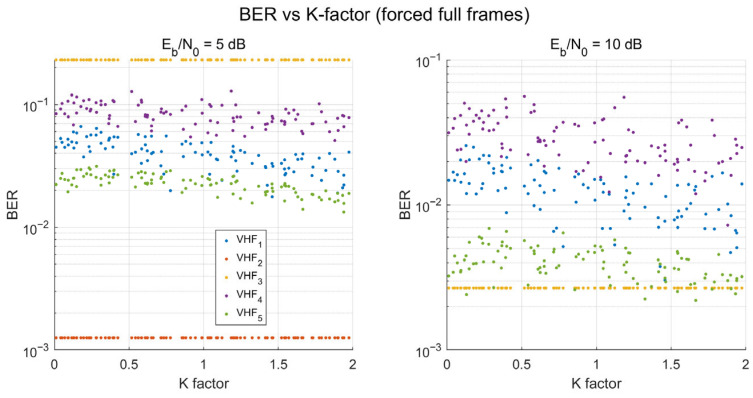
BER vs. K-factor.

K-Factor Uncertainty Analysis. To evaluate robustness against channel model uncertainty in the Rayleigh assumption (K = 0), a Monte Carlo analysis is performed with 1000 independent channel realizations where the Rician K-factor is drawn uniformly from K ∈ [0, 2]. This range spans from pure Rayleigh (K = 0) to mild Rician fading, covering the uncertainty range expected in mountainous terrain, where partial LOS paths may exist intermittently (Table 1). Figure 7 shows the BERs of all five core waveforms at $E_{b}/N_{0}$ = 5 dB and 10 dB.

At both SNR values, VHF_2 (RS + 32FSK) maintains the lowest BER across the entire K-factor range, with minimal sensitivity to K variation. This robustness arises from two factors: non-coherent FSK detection, which is inherently insensitive to the phase characteristics that distinguish Rayleigh from Rician fading; and the RS code’s hard-decision decoding threshold, which remains below the raw error rate regardless of K within this range. The remaining waveforms show moderate BER variation with K but maintain consistent relative ordering. At 10 dB, the separation between waveform clusters increases, providing even more robust selection decisions.

Across 1000 Monte Carlo samples at both SNR points, the optimal selection remains VHF_2, yielding a 0% selection error within the K ∈ [0, 2] uncertainty range. For higher-K environments (e.g., K > 5 dB in maritime or aeronautical scenarios, Table 1), the BER database can be regenerated through the framework’s modular simulation architecture without algorithmic modification.

#### 3.3.4. Simulation Validation

To verify the correctness of the Simulink BER simulation, the simulated BER of VHF_1 (BCH(31,16) + BPSK) is compared against the theoretical union bound under Rayleigh fading. The BCH(31,16) code with error correction capability t = 3 has a theoretical BER upper bound given by:

(11) $$P_{b}\leq \frac{1}{k}\sum_{j=t+1}^{n}j\left(\begin{array}{c} n\\ j \end{array}\right)p^{j}{\left(1-p\right)}^{n-j}$$

where *n* = 31, k = 16, t = 3, and *p* is the uncoded BPSK bit error probability averaged over Rayleigh fading. Table 4 compares the theoretical upper bound with the simulated BER at three representative SNR points within the operational range.

At all three SNR points, the simulated BER falls below the theoretical upper bound, as expected since the union bound is not tight for BCH codes at moderate-to-high SNR. The ratio between the bound and the simulation is approximately 1.7 at both 5 dB and 7 dB, indicating consistent behavior across the SNR range. At 10 dB, no errors were observed at the 10^−6^ detection floor, consistent with the theoretical bound (1.45 × 10^−9^). These results confirm the correctness of the simulation framework, supporting the credibility of the BER characterization for all seven waveform configurations.

## 4. Digital Twin Implementation and Experimental Validation

### 4.1. Simulation Environment Configuration

The digital twin framework is implemented on a single workstation with the following specifications:

Hardware Platform: Intel Core i7-10700 processor @ 2.9 GHz (8 cores, 16 threads), 32 GB DDR4 RAM, Windows 10 operating system. All simulations are executed in single-threaded mode without GPU acceleration.

Software Tools: MATLAB R2023a with Simulink and Communications Toolbox for physical layer simulation; OPNET Modeler 18.0 for network layer protocol simulation; custom C++ SITL interface with WinPcap 4.1.3 for packet capture and injection.

Simulation Performance: Table 5 benchmarks the simulation speed of each waveform configuration on the above platform.

### 4.2. SITL Interface Design

**Figure 8:**
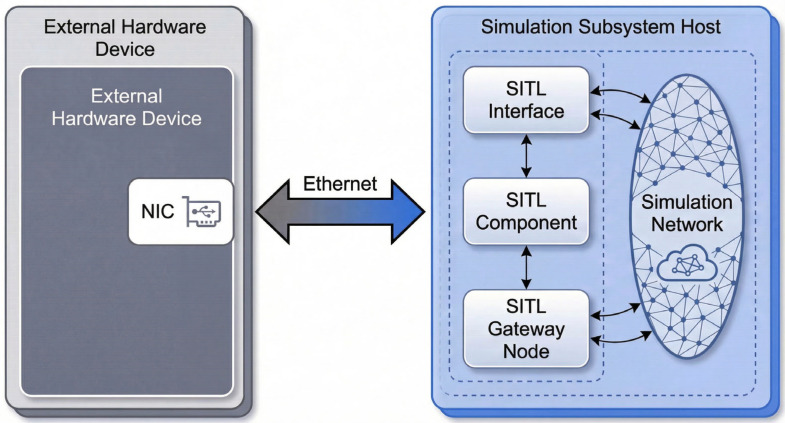
SITL interface architecture.

The Software-In-The-Loop interface enables bidirectional communication between physical VHF equipment and the OPNET simulation environment. Figure 8 shows the interface architecture.

The SITL interface comprises three functional modules. The physical domain interface employs WinPcap for raw packet capture, timestamping, and header parsing. The protocol translation module converts between physical network packet formats and OPNET’s internal representation, maintaining address mapping between physical IPs and simulated node identifiers. The simulation domain interface injects translated packets into the OPNET event queue and extracts simulation-generated packets for reverse translation to physical equipment.

### 4.3. Synchronization Performance

#### 4.3.1. Experimental Setup

Synchronization performance is evaluated through continuous ping tests between physical equipment and virtual nodes. Table 6 summarizes the test configuration.

#### 4.3.2. Synchronization Test Results

Continuous ping tests over 24 h reveal three operating phases. Before simulation activation, the virtual node is inaccessible (destination unreachable). Upon SITL interface startup, round-trip times stabilize at 29–31 ms with mean 30 ms and standard deviation 3.2 ms. Latency breakdown by processing stage: NIC interrupt handling and driver overhead (~5 ms), SITL packet parsing and address mapping (~10 ms), and simulation engine event scheduling (~15 ms). These components were measured by inserting high-resolution timestamps at module boundaries. When simulation pauses and resumes, steady-state latency recovers within 3–4 ping cycles after an initial resynchronization delay.

#### 4.3.3. Delay Impact Analysis

The 30 ms synchronization delay is well within the channel coherence time for low-mobility scenarios ($T_{c}$ ≈ 250 ms at pedestrian speed, 150 MHz) [36], enabling effective real-time state tracking. For vehicular mobility (60 km/h), the 30 ms delay exceeds the coherence time (~22 ms), but the feedback mechanism remains effective for tracking slower-varying interference patterns. In practice, the optimization cycle (~1 s including learning iterations) rather than synchronization delay is the primary latency bottleneck.

### 4.4. Feedback Mechanism Design

#### 4.4.1. Hierarchical Parameter Structure

**Figure 9:**
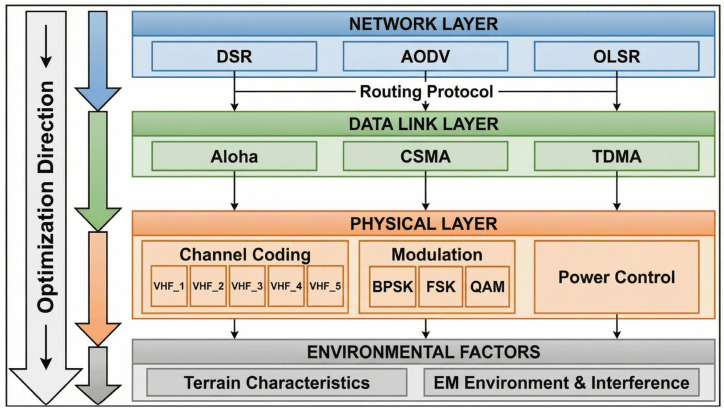
Hierarchical feedback mechanism structure.

Figure 9 illustrates the hierarchical structure of the feedback algorithm, organized according to the OSI layer model adapted for VHF waveform optimization.

Network Layer: Routing protocol selection (DSR, AODV, OLSR) and topology parameters. The current single-hop validation does not exercise the network layer; multi-hop extensions are discussed in Section 4.6.6.

Data Link Layer: MAC protocol type (Aloha, CSMA, TDMA), frame structure, and timing parameters.

Physical Layer: Channel coding scheme (seven waveforms from Section 3), modulation type, and power control parameters.

Environmental Factors: Terrain characteristics and electromagnetic environment including interference levels.

#### 4.4.2. Feedback Algorithm Operation

The feedback algorithm operates continuously to optimize waveform parameters based on real-time conditions. Algorithm 2 presents the detailed procedure.
**Algorithm 2** The steps of proposed digital twin feedback optimization methodCollect real-time data S from physical layer sensors and spectrum monitoring.Update digital twin model parameters to synchronize with physical state.Estimate current channel SNR from sensor measurements by signal processing.Query BER lookup table T to obtain BER values for each candidate waveform at measured SNR.Evaluate performance score $G_{i}$ for each candidate configuration by Equations (7)–(10).Select optimal configuration $X^{*}$ with maximum performance score.Validate configuration through digital twin simulation.Transmit optimized parameters to physical equipment if improvement exceeds threshold ϵ.Return to Step 1.

The general feedback procedure is outlined in Algorithm 2. Its core optimization logic (Steps 5–7) is implemented through the DT-MAB framework detailed in Section 4.6, which replaces the static evaluate-and-select process with continuous online learning.

### 4.5. Hierarchical Performance Evaluation Model

#### 4.5.1. AHP Model Structure

**Figure 10:**
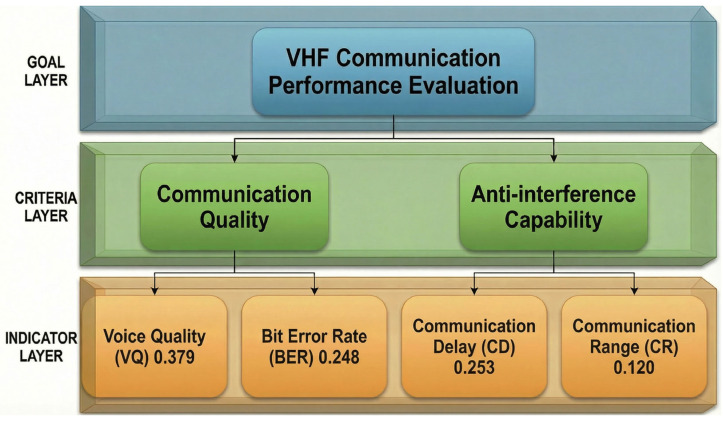
AHP hierarchical evaluation model.

Communication effectiveness is evaluated using the Analytic Hierarchy Process methodology [44]. Figure 10 shows the three-level hierarchy.

Goal Level: Overall VHF communication performance score.

Criteria Level: Communication Quality (successful information transfer factors) and Anti-jamming Capability (interference resilience factors).

Indicator Level: Four measurable indicators—Voice Quality (VQ)/Bit Error Rate (BER)/Communication Delay (CD)/Communication Range (CR).

#### 4.5.2. Expert Consultation Process

The AHP weights are determined through a structured expert consultation process following the methodology of Saaty [44]. Five domain experts (two VHF communication engineers, two military communication specialists, and one academic researcher) provided pairwise comparisons between all indicator pairs using Saaty’s 1–9 scale. Individual judgments were aggregated using the geometric mean method to preserve reciprocity. Table 7 presents the aggregated comparison matrix.

Eigenvector analysis of this matrix yields the following weights:

- Voice Quality: 0.379
- Bit Error Rate: 0.248
- Communication Delay: 0.253
- Communication Range: 0.120

The consistency index CI = 0.024 and consistency ratio CR = CI/RI = 0.024/0.89 = 0.027 < 0.1, confirming acceptable judgment consistency across the expert panel (RI = 0.89 for 4 × 4 matrices per Saaty [44]).

Voice quality receives the highest weight (0.379), reflecting expert consensus that in tactical VHF applications, communication intelligibility directly impacts mission effectiveness and human safety. Delay (0.253) and BER (0.248) have similar weights, as both are critical for reliable data transfer in time-sensitive operations. Range (0.120) has the lowest weight, since it is typically constrained by equipment power specifications and regulatory limits rather than being a primary optimization target.

The effectiveness metric is computed as:

(12) $$G=0.379\cdot {\mathrm{VQ}}_{\mathrm{norm}}+0.248\cdot {\mathrm{BER}}_{\mathrm{norm}}+0.253\cdot {\mathrm{CD}}_{\mathrm{norm}}+0.120\cdot {\mathrm{CR}}_{\mathrm{norm}}$$

where indicator values are normalized to a [0, 1] range before aggregation.

#### 4.5.3. Weight Robustness Analysis

To validate the robustness of the AHP-derived weights, we perform a sensitivity analysis by perturbing each weight by ±20% (with the remaining weights renormalized to preserve unit sum), generating 9 weight configurations: the baseline plus 4 weights × 2 perturbation directions. For each perturbed weight set, we recompute the AHP score G across all 63 candidate configurations and rank them.

The analysis reveals strong robustness of the optimization outcome to weight perturbations. The Top-3 configuration set—{arm 27 (VHF_3 + TDMA + 20 W), arm 63 (VHF_7 + TDMA + 20 W), arm 36 (VHF_4 + TDMA + 20 W)}—remains invariant across all 9 weight perturbation scenarios. Within this set, the rank ordering between the top two configurations exchanges in two out of nine scenarios, with G-score differences below 0.005 in those exchange cases—well within the noise floor of any practical decision system. The remaining 7 scenarios preserve the baseline rank ordering exactly.

This result confirms that the optimization conclusions are not artifacts of a particular AHP weight choice within plausible expert-judgment ranges. Combined with the consistency ratio CR = 0.027 (well below Saaty’s 0.1 threshold) reported in Section 4.5.2, the weight design is both internally consistent and externally robust.

### 4.6. Online Learning-Based Adaptive Optimization

The preceding sections established the BER performance database (Section 3.3), the SITL synchronization mechanism (Section 4.2 and Section 4.3), and the AHP-weighted evaluation model (Section 4.5). This section introduces the core optimization algorithm that leverages these components: a contextual multi-armed bandit (MAB) framework with digital twin-enabled virtual exploration, referred to as DT-MAB.

#### 4.6.1. Problem Formulation

The adaptive waveform selection problem is formulated as a contextual multi-armed bandit problem. At each decision cycle *t*, the system observes a context vector $x_{t}\in {\mathbb{R}}^{4}$ provided by the digital twin:

(13) $$x_{t}={\left[{\mathrm{SNR}}_{t},\;I_{t},\;f_{d,t},\;\eta_{t}\right]}^{T}$$

where ${\mathrm{SNR}}_{t}$ (dB) is the measured signal-to-noise ratio, $I_{t}$ (dB) is the estimated interference power level, $f_{d,t}$ (Hz) is the Doppler spread estimate, and $\eta_{t}$ ∈ [0, 1] is the MAC-layer load factor. These four quantities are extracted from the physical layer measurement vector $y_{p}(t)$ introduced in Section 2.2.7.

The action space comprises K = 63 candidate configurations, formed by the Cartesian product of three optimization dimensions:

- Waveform: W∈{1,2,…,7} (VHF_1 through VHF_7 as defined in Table 2)
- MAC protocol: Z∈{1,2,3} (Aloha, CSMA, TDMA)
- Transmit power level: P∈{1,2,3} (5 W, 10 W, 20 W)

Each action (arm) k corresponds to a unique triplet ($W_{k}$, $Z_{k}$, $P_{k}$). The reward $r_{t}\in [0,1]$ observed after deploying arm k at time t is the AHP-weighted performance score G computed by Equation (12), evaluated from physical layer feedback measurements. The current reward formulation prioritizes communication performance (voice quality, BER, delay, range) and does not include an explicit energy term; the de facto energy behavior under this formulation and a mechanism for incorporating explicit energy-awareness are analyzed in Section 4.7.5.

The objective is to maximize the cumulative reward over T decision cycles, or equivalently to minimize the cumulative regret:

(14) $$R(T)=\sum_{t=1}^{T}\left[G^{*}(x_{t})-G_{k_{t}}(x_{t})\right]$$

where $G^{*}(x_{t})$ is the reward of the oracle-optimal arm for context $x_{t}$, and $G_{k_{t}}(x_{t})$ is the reward of the arm $k_{t}$ selected at time t.

#### 4.6.2. Lin-UCB Algorithm with Virtual Exploration

We adopt the Linear Upper Confidence Bound (Lin-UCB) algorithm [45] as the learning engine, which models the expected reward of each arm k as a linear function of the context vector:

(15) $$E\left[r_{t}|x_{t},k\right]=\theta_{k}^{T}\cdot x_{t}$$

where $\theta_{k}\in {\mathbb{R}}^{4}$ is the unknown parameter vector for arm k. Lin-UCB maintains, for each arm k, a matrix $A_{k}\in {\mathbb{R}}^{4\times 4}$ and a vector $b_{k}\in {\mathbb{R}}^{4}$ that accumulate historical observations:

(16) $$A_{k}=I_{d}+\sum_{\tau :k_{\tau }=k}x_{\tau }x_{\tau }^{T},\;b_{k}=\sum_{\tau :k_{\tau }=k}r_{\tau }x_{\tau }$$

The parameter estimate and upper confidence bound are computed as:

(17) $${\hat{\theta }}_{k}=A_{k}^{-1}\cdot b_{k}$$

(18) $${\mathrm{UCB}}_{k}={\hat{\theta }}_{k}^{T}\cdot x_{t}+\alpha \sqrt{x_{t}^{T}\cdot A_{k}^{-1}\cdot x_{t}}$$

where α>0 controls the exploration–exploitation trade-off. The first term represents the estimated reward (exploitation), while the second term quantifies estimation uncertainty (exploration). Arms with high estimated reward or high uncertainty receive higher UCB scores, encouraging the algorithm to explore promising but under-sampled configurations.

A critical challenge arises when deploying MAB in safety-critical VHF communication systems: exploring an untested configuration on the physical link may cause severe performance degradation, including communication loss during tactical operations. To address this, we introduce a virtual exploration mechanism that leverages the digital twin as a pre-deployment evaluation sandbox.

When Lin-UCB selects a candidate arm k*, the digital twin first simulates the expected performance $G_{\mathrm{virtual}}$ by evaluating arm k* under the current context $x_{t}$ using the BER lookup table (Table 3) and the AHP evaluation model (Equation (8)). If $G_{\mathrm{virtual}}$ falls below the current best-known performance $G_{\mathrm{current}}^{*}$ by more than a safety threshold ε, the system reverts to the current best arm rather than risking the physical link:

(19) $$k_{\mathrm{deploy}}=\left\{\begin{array}{ll} k^{*}, & \mathrm{if}\;G_{\mathrm{virtual}}\geq G_{\mathrm{current}}^{*}-\varepsilon \\ k_{\mathrm{exploit}}, & \mathrm{otherwise} \end{array}\right.$$

where $k_{\mathrm{exploit}}=\arg\max_{k}\;{\hat{\theta }}_{k}^{T}\cdot x_{t}$ is the greedy exploitation choice. This mechanism ensures that physical link disruptions caused by exploratory actions are bounded while still allowing the algorithm to explore promising configurations through the digital twin’s virtual evaluation.

The complete DT-MAB procedure is formalized in Algorithm 1.

The parameter α = 0.5 and threshold ε = 0.05 are selected through grid search over α∈{0.1,0.3,0.5,0.8,1.0} and ε∈{0.01,0.03,0.05,0.10}, evaluated on 10 independent simulation runs of 500 decision cycles each. The combination (α=0.5, ε=0.05) achieved the lowest average cumulative regret while maintaining fewer than 5 virtual exploration rejections per 100 cycles, indicating a balanced exploration–exploitation trade-off.

**Figure 11:**
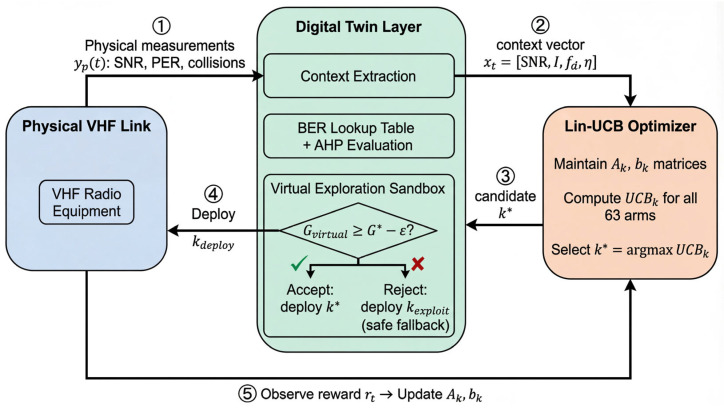
DT-MAB decision cycle for adaptive waveform selection.

Figure 11 illustrates the DT-MAB decision cycle. The closed-loop operation proceeds in five numbered steps: ① the physical VHF link reports measurements $y_{p}(t)$ to the digital twin; ② the digital twin extracts the context vector $x_{t}$ and forwards it to the Lin-UCB optimizer; ③ Lin-UCB computes UCB scores for all 63 arms and outputs the candidate $k^{*}$; ④ the virtual exploration sandbox within the digital twin evaluates $k^{*}$ against the safety criterion $G_{\mathrm{virtual}}\geq G_{\mathrm{current}}^{*}-\varepsilon$—accepting k if the condition holds or falling back to the safe exploitation choice $k_{\exp loit}$ otherwise—and deploys the final selection $k_{deploy}$ to the physical link; ⑤ the observed reward r_t from the physical link is fed back to update the Lin-UCB model parameters $A_{k}$ and $b_{k}$. This architecture ensures that exploratory actions are screened by the digital twin before affecting the physical communication link.

#### 4.6.3. Role of the Digital Twin in Online Learning

The digital twin serves two distinct and indispensable functions within the DT-MAB framework, beyond its conventional role as a simulation platform:

Function 1: Context provision. The digital twin continuously processes raw physical measurements $y_{p}(t)$ into the structured context vector $x_{t}$ required by Lin-UCB. This involves signal processing operations—SNR estimation from received signal strength, interference level extraction through spectral analysis, Doppler estimation from channel autocorrelation, and MAC load computation from collision statistics—that are performed within the digital layer’s data processing module (Figure 1). Without the digital twin, these context features would need to be computed by the resource-constrained VHF equipment itself.

Function 2: Virtual exploration sandbox. As formalized in Step 4 of Algorithm 1, the digital twin evaluates candidate configurations before physical deployment, preventing potentially harmful exploratory actions from disrupting the communication link. This function is unique to the digital twin architecture and cannot be replicated by an offline optimization approach or a standalone MAB algorithm. The quantitative impact of this mechanism is validated through ablation experiments in Section 4.7.4.

The combination of these two functions transforms the digital twin from a passive monitoring tool into an active participant in the optimization loop. Removing either function—as demonstrated in the MAB-only ablation baseline (Section 4.7.4)—results in measurable performance degradation.

#### 4.6.4. Relationship to Prior PSO-Based Optimization

The previously reported PSO-based optimizer [46] is retained as a comparison baseline (Section 4.7.4). The key distinction is the learning paradigm: PSO performs independent optimization each cycle without cross-cycle memory, whereas Lin-UCB accumulates knowledge through $A_{k}$ and $b_{k}$ for progressively better decisions. PSO with N = 10 particles requires ~87 function evaluations per cycle, comparable to Lin-UCB’s 63 UCB computations plus at most one digital twin simulation. A more comprehensive complexity analysis covering memory usage and multi-node scalability is presented in Section 4.6.6.

#### 4.6.5. Theoretical Justification for the Digital Twin’s Benefit

A natural concern is whether the digital twin actually improves the cumulative reward G or may inadvertently degrade it through imperfect virtual evaluation. The framework provides theoretical and empirical safeguards on three levels. First, the virtual exploration sandbox formalized in Equation (19) acts as a conservative filter: only candidate configurations satisfying $G_{virtual}\geq G_{current*}-\varepsilon$ are deployed. Otherwise, the system reverts to the safe exploitation choice $k_{\exp loit}$. This guarantees that, under unbiased predictions, the deployed action’s expected reward is at least $G_{current*}-\varepsilon$ (with ε = 0.05 in our experiments), so the digital twin can only improve over the no-DT baseline in expectation, never degrade. Second, prediction fidelity is supported by the 30 ms SITL synchronization (Section 4.3) keeping the digital twin’s state within the channel coherence time, while the safety margin ε absorbs small prediction errors without rejecting marginally beneficial actions. Third, the ablation experiment in Section 4.7.4 empirically confirms the theoretical guarantee: removing the virtual exploration mechanism increases mean cumulative regret by 41% (5645 → 7951) and performance drop events by 49% (250 → 373) across 10 seeds, demonstrating that the digital twin’s contribution is structurally guaranteed, conditionally protected, and substantial in magnitude.

#### 4.6.6. Scalability and Computational Complexity

The DT-MAB framework’s per-cycle computational cost comprises three components: Lin-UCB optimization, digital twin virtual exploration, and SITL synchronization. Table 8 summarizes the complexity and measured timing for the current 63-arm configuration.

The Lin-UCB step computes UCB scores for all K arms, requiring matrix inversion $A_{k^{-1}}\in R^{\left(d\times d\right)}$ for each arm. The total complexity is $O(K\cdot d^{3})=O(63.64)\approx 4000$ operations per cycle, completing in under 1 ms on the workstation specified in Section 4.1. Memory requirements are negligible. Each arm maintains $A_{k}(d\times d)$ and $b_{k}(d\times 1)$, totaling $K\cdot \left(d^{2}+d\right)=1260$ floating-point values (approximately 10 KB). The digital twin virtual exploration involves at most one Simulink simulation call (~100 ms for the dominant Turbo decoder; faster for other waveforms per Table 5). Combined with the 30 ms SITL synchronization (Section 4.3), the total per-cycle decision time is below 105 ms, providing over 9× headroom relative to the 1-s decision interval. The actual scalability bottleneck is therefore the digital twin simulation cost, not the bandit algorithm or memory storage.

For multi-node tactical networks, the action space scales as $K^{N}$ for N nodes under joint optimization, making direct extension infeasible beyond approximately 3–5 nodes (e.g., $63^{5}\approx 10^{9}$ arms exceeds the 1-s budget by orders of magnitude). Three practical strategies can address larger networks:

(1) Hierarchical decomposition. Each node runs an independent DT-MAB instance with periodic coordination at a slower timescale (e.g., every 10 s for topology-level decisions). This preserves the per-node action space at K = 63 and supports networks of arbitrary size at the cost of suboptimality relative to joint optimization.
(2) Action space pruning. Pareto-optimal subsets of the joint action space can be identified offline through systematic BER–MAC–power trade-off analysis, restricting online exploration to typically 5–10× fewer configurations than the full Cartesian product. The framework’s modular waveform database (Section 3.1) supports such pruning without algorithmic modification.
(3) Federated learning of the parameter matrices $A_{k}$, $b_{k}$ across nodes accelerates convergence by leveraging the cross-node correlation in channel and traffic statistics, particularly relevant for geographically clustered tactical units operating in similar electromagnetic environments.

The current single-hop point-to-point validation (Section 4.7.1) establishes the foundational framework. Multi-node extensions through the above strategies are identified as future work (Section 5.3).

### 4.7. Experimental Results

#### 4.7.1. Experimental Scenario Configuration

**Figure 12:**
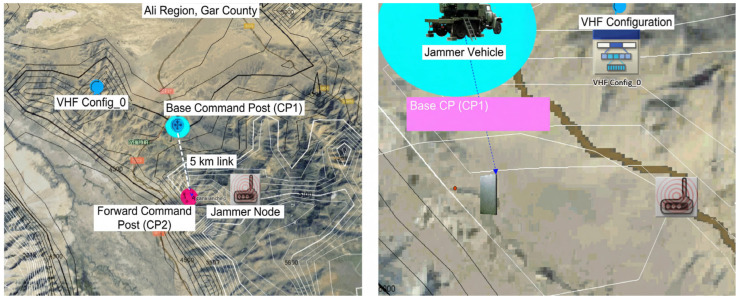
Experimental scenario configuration: terrain overview, node deployment, and jammer vehicle trajectory in a mountainous valley (Ali region, elevation 4300–5000 m), with the 5 km VHF link between the Base Command Post (CP1) and Forward Command Post (CP2).

The experimental validation is conducted within a simulated mountainous tactical scenario representative of ground-mobile VHF operations. Figure 12 shows the scenario geometry overlaid on terrain elevation data.

Terrain Environment: The scenario models a narrow valley with elevation variations typical of high-altitude border regions. The digital terrain elevation model is imported into OPNET Modeler to ensure realistic propagation path modeling. Low ridgelines exist between the two communication endpoints but do not obstruct the first Fresnel zone at VHF frequencies, maintaining a marginal line-of-sight path consistent with the Rayleigh fading assumption discussed in Section 2.2.1.

Communication Link Geometry: A 5 km point-to-point VHF link connects a base command post (BCP) and a forward command post (FCP), representing a typical tactical communication relay segment. Table 9 summarizes the link parameters.

Link Budget: The 5 km link at 30.525 MHz provides an approximately 63 dB margin above receiver sensitivity under free-space conditions. With Rayleigh fading, the instantaneous SNR fluctuates between 10 and 75 dB under the configured jammer trajectory (illustrated in Section 4.7.3), with the lower bound representing deep fade events.

Jammer Configuration: An interference source is positioned near the forward command post to simulate adversarial electronic warfare. Table 9 details the jammer parameters.

**Figure 13:**
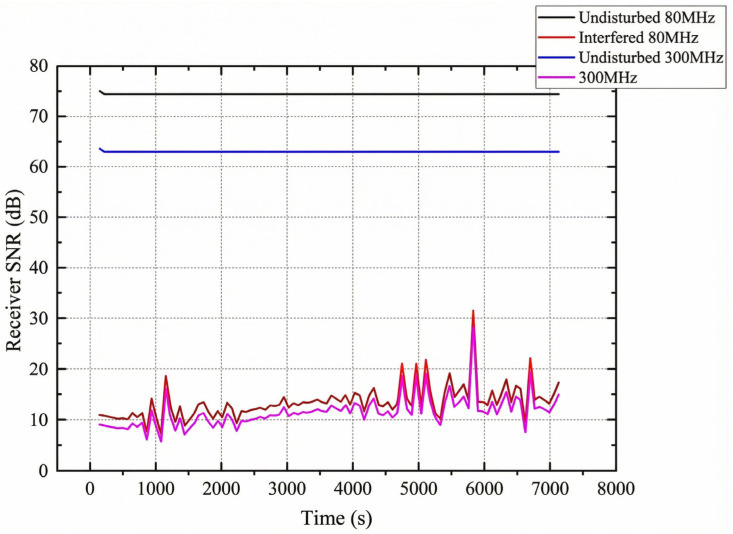
SNR comparison with and without interference.

Jammer Trajectory: The interference node traverses a linear path directly through the BCP-FCP link midpoint over approximately 74 min, producing time-varying interference power at the receiver. This geometry creates the SNR fluctuations visible in Figure 13, enabling evaluation of the digital twin’s adaptation under dynamically changing conditions.

#### 4.7.2. Protocol Switching Validation

**Figure 14:**
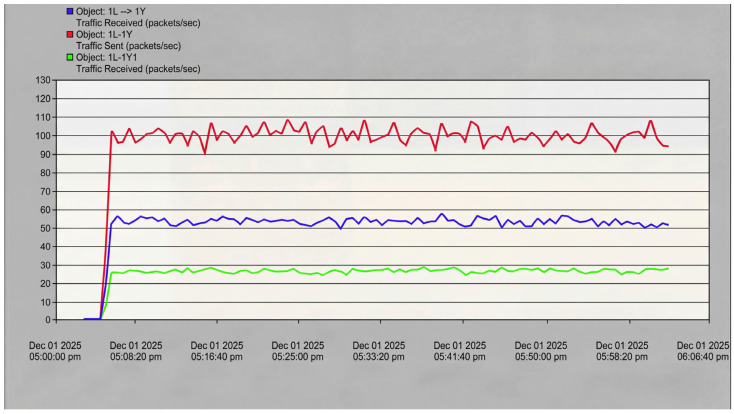
Traffic statistics for protocol switching experiment.

The first experiment validates feedback-based protocol switching. A multi-node VHF network operates initially with Aloha MAC, exhibiting ~17% packet loss at ~100 packets/s aggregate rate (Figure 14), which is consistent with Aloha’s theoretical 18.4% maximum throughput limit. Digital twin analysis identifies this congestion and recommends switching to CSMA, after which packet loss decreases to 8% (53% relative improvement).

#### 4.7.3. Interference Characterization

The interference characterization uses the scenario in Section 4.7.1 (Table 9). Preliminary tests across 30/80/300 MHz jammer frequencies confirmed that propagation loss dominates over frequency-dependent effects for narrowband VHF interference. Subsequent experiments focus on the 30 MHz in-band configuration.

SNR Degradation Analysis: Figure 13 compares SNR with and without interference.

Non-interfered operation maintains a stable SNR of 65–75 dB. Under 30 W interference, SNR drops to 10–25 dB (~50 dB degradation), corresponding to the medium-performance region of the BER curves (Figure 3, Table 3) where waveform selection has the greatest impact.

**Figure 15:**
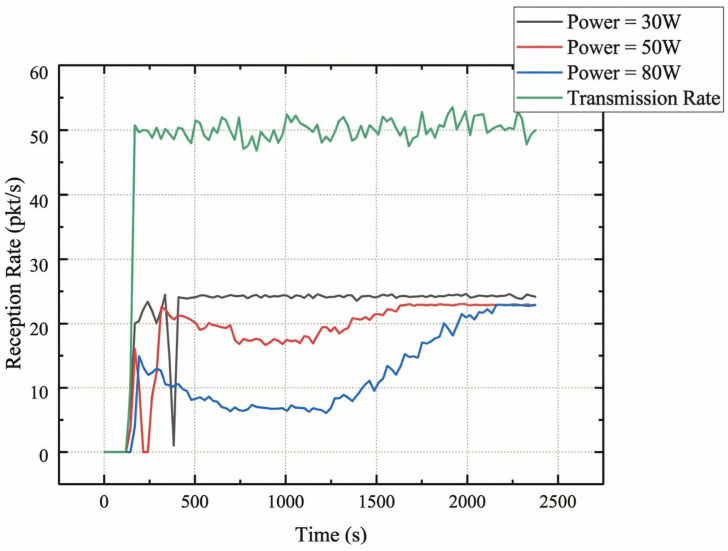
Reception rate versus interference power.

Reception Rate Impact: Figure 15 quantifies the relationship between interference power and reception performance.

Table 10 summarizes reception performance across four interference levels. The nonlinear power-to-loss relationship reflects the BER-SNR waterfall: Small SNR reductions in the 3–10 dB region produce large BER increases. The 30 W scenario—maintaining a degraded but functional link—is selected as the primary test condition for Section 4.7.4, providing headroom for waveform-level optimization. The 300 W denial-of-service scenario requires frequency hopping or spatial countermeasures beyond waveform selection (Section 5.3).

#### 4.7.4. DT-MAB Online Learning Performance

To validate the proposed DT-MAB framework (Section 4.6), a comprehensive experiment is conducted under the same mountainous tactical scenario described in Section 4.7.1. The experiment spans 500 decision cycles (1 s per cycle) and is divided into three phases to evaluate adaptation under changing conditions:

- Phase 1 (Rounds 1–200): Normal operation without interference, allowing the learning algorithm to converge.
- Phase 2 (Rounds 201–400): 30 W narrowband jamming is activated (Table 9 configuration), creating an abrupt environment change.
- Phase 3 (Rounds 401–500): Jamming is deactivated, testing recovery speed.
- Six methods are compared:
- Oracle: Selects the globally optimal configuration at each round with perfect channel knowledge (theoretical upper bound, not achievable in practice).
- DT-MAB: The proposed framework (Algorithm 1) with α = 0.5, ε = 0.05.
- MAB-only: Lin-UCB without digital twin virtual exploration (ablation baseline; Step 4 of Algorithm 2 is bypassed, and $k^{*}$ is deployed directly).
- DT-PSO: The original PSO-based optimizer [46] with digital twin evaluation (ω = 0.7, $c_{1}=c_{2}=1.5$, N=10 particles).
- Rule-Based: SNR threshold-based waveform switching (switch to VHF_2 when measured SNR < 15 dB).
- Fixed: Static VHF_1 (BCH + BPSK) with CSMA and 5 W power throughout.

All methods operate on the expanded 63-configuration space (7 waveforms × 3 MAC protocols × 3 power levels) and use the AHP-weighted score G (Equation (8)) as the performance metric. Each experiment is repeated with 10 independent random seeds for channel fading realization, and results are averaged.

**Figure 16:**
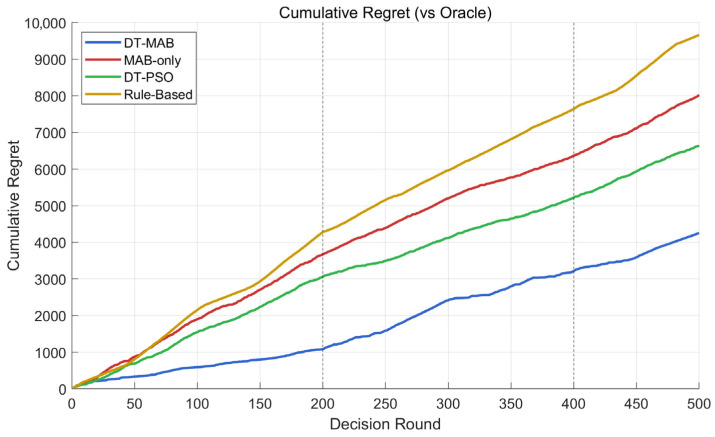
Cumulative regret comparison of four adaptive methods over 500 decision rounds. Vertical dashed lines indicate jamming onset (Round 200) and cessation (Round 400).

Cumulative Regret Analysis. Figure 16 presents the cumulative regret $R(t)=\sum_{\tau =1}^{t}\left[G^{*}(x_{\tau })-G_{k_{\tau }}(x_{\tau })\right]$ for four adaptive methods over 500 decision rounds. The fixed configuration baseline is omitted from this plot as its regret grows linearly and would compress the visual scale of the other curves.

DT-MAB achieves the lowest mean cumulative regret across 10 seeds (5645 ± 1621), outperforming DT-PSO (6762 ± 131) by 16.5% and MAB-only (7951 ± 112) by 29.0% on average; DT-MAB outperforms DT-PSO in 7/10 seeds, with the remaining 3 seeds showing comparable performance. The substantially higher variance of DT-MAB reflects the path-dependent nature of online learning in a 63-arm action space: across all 10 seeds, DT-MAB consistently identifies VHF_1 + TDMA as the dominant policy family in Phase 1 (varying only in power level: 5/10/20 W across seeds), but the specific arm within this family and subsequent Phase 3 convergence target depends on early exploration randomness. This is a fundamental characteristic of contextual bandits in large action spaces—the algorithm reliably learns the structure of the optimal policy while exhibiting natural variability in its realization.

The three-phase regret growth visible in Figure 16 reveals mechanistic differences between methods. DT-MAB exhibits near-flat regret growth in Phase 1 after the initial learning period (within ~50 rounds), accelerated growth during Phase 2 jamming, and recovery of low-slope behavior in Phase 3. DT-PSO accumulates regret steadily across all phases due to its lack of cross-cycle memory. MAB-only suffers from unscreened exploratory actions that occasionally cause performance drops. Rule-based switching shows the highest regret slope as it cannot adapt beyond a fixed SNR threshold rule.

**Figure 17:**
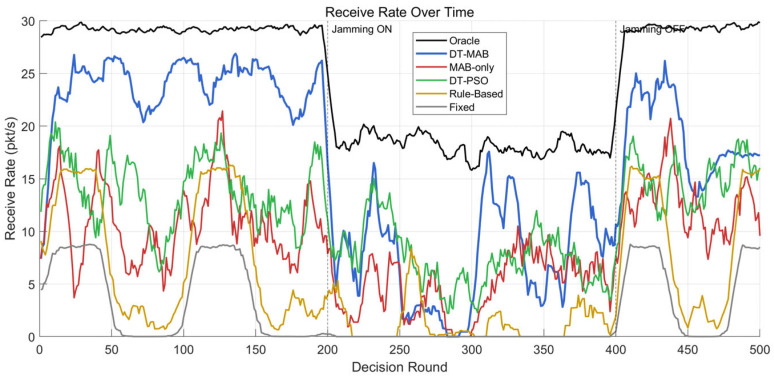
Reception rate over 500 decision rounds for six methods under three-phase scenario (no jamming → 30 W jamming → recovery).

Reception Rate Comparison. Figure 17 shows the instantaneous reception rate (packets/s) over time for all six methods. Table 11 summarizes the phase-averaged performance.

The phase-averaged reception rates display three regimes corresponding to the experimental phases. In Phase 1 (no jamming), DT-MAB outperforms DT-PSO (13.6 ± 0.3 pkt/s) by 25% and MAB-only (11.2 ± 0.3 pkt/s) by 52%, demonstrating the combined benefit of online learning and digital twin virtual exploration under stable conditions. The fixed configuration yields only 3.8 pkt/s, confirming that the default VHF_1 + CSMA + 5 W is far from optimal even without interference.

In Phase 2 (30 W jamming), all methods experience severe degradation. The Oracle drops from 29.2 to 18.0 pkt/s, reflecting the physical limits of interference suppression even with optimal configuration and maximum power. DT-MAB (8.7 ± 1.8 pkt/s) and DT-PSO (7.5 ± 0.6 pkt/s) perform comparably because both converge to high-power configurations (74% and 67% selection of 20 W respectively), reflecting that maximum power is necessary under strong interference and reducing the relative advantage of online learning. Both digital twin-assisted methods substantially outperform MAB-only (4.5 ± 0.2 pkt/s) and Rule-Based (1.5 ± 0.2 pkt/s), confirming the value of digital twin pre-evaluation under degraded conditions.

In Phase 3 (recovery), DT-MAB regains its advantage over DT-PSO by 16%, leveraging cross-phase memory accumulated in the $A_{k}$ and $b_{k}$ matrices for faster re-adaptation; DT-PSO must re-optimize from scratch at each cycle. An interesting structural observation emerges from per-seed analysis: 6 out of 10 seeds exhibit a configuration switch between Phase 1 and Phase 3, with post-jamming dominant arms shifting to VHF_3, VHF_4, or VHF_7 variants depending on seed-specific exploration history. This perturbation-driven re-exploration is a robust mechanism (60% occurrence rate) and reflects the existence of multiple near-optimal configurations in the 63-arm space (also visible in Oracle’s diffuse selection pattern: the Top-1 arm captures only 20–37% of selections per phase).

**Figure 18:**
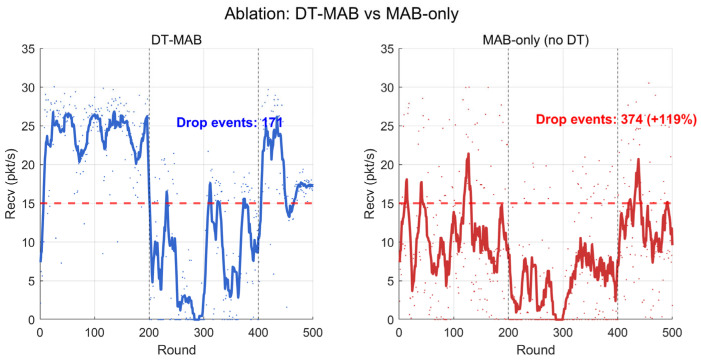
Ablation comparison: DT-MAB (**left**, blue trace) versus MAB-only without digital twin (**right**, red trace). The red dashed line indicates the 15 pkt/s performance threshold. Numbers in the plots indicate total drop events below this threshold.

Ablation Study. To isolate the contribution of the digital twin’s virtual exploration mechanism, we compare DT-MAB with MAB-only, which uses identical Lin-UCB parameters but bypasses Step 4 of Algorithm 1 (virtual pre-evaluation). Figure 18 visualizes the reception rate traces of both methods, with performance drop events (reception rate < 15 pkt/s) highlighted (Table 12).

The ablation results quantify two complementary contributions of the digital twin. First, the virtual exploration sandbox reduces performance drop events by 33% (250 vs. 373, *p* < 0.01), as DT-MAB rejects the deployment of configurations whose simulated reward falls below the safety threshold  ε=0.05 (Equation (19)). Second, by providing the structured context vector $x_{t}$ extracted from raw measurements, the digital twin enables Lin-UCB to operate on a well-conditioned 4-dimensional feature space, accelerating convergence to the dominant policy family (within approximately 50 rounds in Phase 1).

Quantitatively, removing the digital twin increases mean cumulative regret by 41% (from 5645 to 7951) and performance drop events by 49% (from 250 to 373), confirming that the digital twin is a functionally indispensable component of the online learning architecture rather than a system integration convenience.

#### 4.7.5. Energy Efficiency Analysis

Tactical VHF radios are typically battery-powered, making energy efficiency a practical concern for deployment. The DT-MAB framework includes transmit power as one of three optimization dimensions (5/10/20 W), but the AHP-weighted reward G (Equation (12)) does not include an explicit energy term. This subsection analyzes the de facto energy behavior of DT-MAB under the current reward design and discusses how explicit energy-awareness can be incorporated.

Across 10 seeds, DT-MAB selects the 20 W power level in 46.1 ± 39.4% of Phase 1 rounds, 60.0 ± 13.9% of Phase 2 rounds, and 69.9 ± 16.8% of Phase 3 rounds, yielding a mean transmit power of 14.36 ± 3.01 W. The high cross-seed variance in Phase 1 power selection (σ = 39.4%) reflects the exploration-dependent learning trajectory characteristic of contextual bandits in large action spaces (Section 4.7.4): Some seeds converge to high-power configurations within the VHF_1 + TDMA family while others discover lower-power alternatives that remain competitive in reward. This emergent diversity in power selection contrasts with the more deterministic behavior of DT-PSO (mean 15.02 ± 0.18 W) and Oracle (14.30 ± 0.19 W), both of which exhibit very low cross-seed variance because their decisions are not history-dependent.

Table 13 compares mean transmit power and energy efficiency (defined as packets received per Watt-second of transmit energy) across the six methods.

Two observations emerge from Table 13. First, DT-MAB’s mean transmit power (14.36 W) is comparable to DT-PSO (15.02 W) and Oracle (14.30 W), and lower than the upper power range that one might naïvely expect from a performance-prioritized reward. This is a non-trivial result: the Lin-UCB algorithm autonomously discovers that maximum power is not always required to maximize the reward G, since at sufficient SNR the BER, voice quality, and range indicators saturate and additional power yields diminishing returns. Second, DT-MAB achieves the highest energy efficiency among all six methods (0.933 pkt/(s·W)), exceeding Rule-Based (0.880), DT-PSO (0.747), and MAB-only (0.680). The Oracle baseline reaches 1.731 pkt/(s·W), defining the theoretical upper bound for the action space. This finding indicates that online learning not only maximizes the reception rate but also implicitly identifies energy-efficient operating points that static or single-cycle methods cannot reach.

While the current reward formulation already produces favorable energy behavior, deployment scenarios with strict energy budgets (e.g., long-duration covert operations, battery-limited unmanned platforms) may require explicit control over the performance-energy trade-off. The reward function can be augmented with an explicit energy penalty:

(20) $$G_{energy}=G-\lambda \cdot \left(P_{tx}/P_{\max}\right)$$

where λ ≥ 0 controls the performance-energy trade-off and $P_{\max}$ = 20 W is the maximum power level. This extension requires no modification to the Lin-UCB algorithm, the digital twin architecture, or the 63-configuration action space; only the reward computation in Algorithm 1 (Step 13) needs adjustment. With λ = 0, the system reverts to the current behavior; for λ→∞, the system minimizes power consumption regardless of link quality. Intermediate values produce Pareto-frontier solutions trading reception rate against energy consumption.

Systematic evaluation of energy-aware variants under different λ values, including derivation of operationally meaningful λ from mission energy budgets, is identified as future work (Section 5.3).

## 5. Conclusions

### 5.1. Summary of Contributions

This paper presented a digital twin-enabled online learning framework (DT-MAB) for adaptive waveform selection in VHF tactical communication systems. The framework addresses the limitations of static optimization approaches by introducing continuous online learning through contextual multi-armed bandits, with the digital twin serving as a virtual exploration sandbox that ensures deployment safety.

The SITL-based synchronization mechanism achieved 30 ms average latency with 3.2 ms standard deviation, enabling real-time interaction between the physical and virtual domains. Systematic BER characterization of seven waveform configurations established the performance database, with a comparative analysis confirming the design rationale for the core configuration set: Turbo + 16QAM (VHF_6) was shown to be inferior to Turbo + BPSK (VHF_5) under Rayleigh fading due to amplitude estimation sensitivity and RS + BPSK (VHF_7) was shown to be inferior to RS + 32FSK (VHF_2) due to symbol-modulation misalignment. Robustness analyses demonstrated that waveform selection decisions remain stable across Doppler frequencies from 5 to 20 Hz and Rician K-factor variations from 0 to 2, with zero selection errors in 1000 Monte Carlo trials.

The DT-MAB framework demonstrated robust advantages across 10 random seeds in the 500-round three-phase evaluation, achieving the lowest mean cumulative regret (5645 ± 1621), reducing regret relative to DT-PSO (6762 ± 131) by 16.5%, relative to MAB-only (7951 ± 112) by 29.0%, and relative to rule-based switching (9607 ± 57) by 41.2%; DT-MAB outperformed DT-PSO in 7/10 seeds. The protocol switching experiment validated the feedback mechanism, reducing packet loss from 17% to 8% through intelligent Aloha-to-CSMA transition. Ablation experiments confirmed the digital twin’s indispensable role: Removing the virtual exploration mechanism increased mean performance drop events by 49% and cumulative regret by 41%, demonstrating that the digital twin is a functionally necessary component of the online learning architecture rather than a system integration convenience.

### 5.2. Limitations

Several limitations should be acknowledged. First, the physical layer BER characterization employs Rayleigh fading for the target mountainous tactical scenario, with Rician fading analysis (K = 5 dB) confirming framework extensibility to LOS environments. Other VHF environments (Table 1) would require scenario-specific calibration. RF front-end non-idealities are not explicitly modeled; the estimated 1–3 dB implementation loss is expected to preserve relative waveform rankings, but field validation with operational VHF equipment remains essential.

Second, while DT-MAB achieves comparable performance to DT-PSO during active jamming (8.7 vs. 7.5 pkt/s in Phase 2), both digital twin-assisted methods experience significant degradation relative to Phase 1, indicating that waveform-level optimization alone cannot fully compensate for strong interference. For scenarios with very rapid environment transitions, a hybrid approach combining rule-based fast response with DT-MAB continuous optimization is identified as future work.

Third, scalability is currently validated only for the single-hop point-to-point link described in Section 4.7.1. Direct extension to multi-node tactical networks (joint optimization) is constrained by the $K^{N}$ action space scaling, with practical centralized limits of approximately 3–5 nodes. Distributed strategies—hierarchical decomposition, Pareto-pruned action spaces, or federated parameter learning across nodes (Section 4.6.6)—can scale to arbitrary network sizes but introduce coordination overhead and may sacrifice global optimality. Empirical validation of these multi-node strategies is identified as future work.

Fourth, the AHP-weighted reward function (Equation (12)) does not include an explicit energy term. While the analysis in Section 4.7.5 shows that DT-MAB nonetheless achieves the highest energy efficiency among all evaluated methods (0.933 pkt/(s·W)), this favorable behavior emerges implicitly from the reward saturation properties at sufficient SNR rather than from explicit optimization. Deployments with strict energy budgets—where energy consumption must be controlled directly rather than as a side effect—would benefit from the energy-aware reward extension proposed in Section 4.7.5 (Equation (20)).

Fifth, the interference model is limited to single-source narrowband continuous-wave jamming with a predetermined linear trajectory. Practical contested environments may involve wideband jamming, frequency-hopping interference, or multiple coordinated jammers. The 300 W denial scenario (Table 10) confirmed that waveform-level optimization alone cannot overcome extreme power disadvantages.

Sixth, experimental validation uses software simulation and SITL hardware emulation rather than deployed VHF equipment. Real-world propagation effects including terrain-dependent multipath, atmospheric variations, and equipment aging may affect performance in ways not captured by the current models.

### 5.3. Future Work

Future research directions include:

1. Hardware-in-the-loop validation: Integrating the digital twin framework with operational VHF transceivers to validate BER predictions against over-the-air measurements, calibrate simulation models with real-world channel statistics, and incorporate parametric RF front-end models (PA nonlinearity, phase noise) to close the 1–3 dB fidelity gap.
2. Advanced interference scenarios: Extending the framework to handle wideband interference, intelligent adaptive jamming, and multi-jammer scenarios through expanded waveform databases and coordinated multi-node optimization.
3. Advanced learning algorithms: Extending the Lin-UCB framework to deep contextual bandits or deep reinforcement learning for larger action spaces and nonlinear context–reward relationships, with hybrid fast-slow adaptation combining rule-based immediate response for abrupt changes with continuous MAB optimization for steady-state performance.
4. Multi-node and large-scale deployment: Extending the framework to multi-hop tactical networks through the scalability strategies discussed in Section 4.6.6 (hierarchical decomposition, action space pruning, federated learning of bandit parameters). Empirical evaluation of trade-offs between coordination overhead and global optimality across these strategies is a key open question.
5. Energy-aware reward extension and Pareto-frontier characterization: Implementing the energy-augmented reward $G_{energy}$ (Equation (20)) and characterizing the performance-energy Pareto frontier across operationally meaningful λ values. Although the current performance-only reward already yields the highest energy efficiency among the compared methods (Section 4.7.5), explicit energy control is required for deployment scenarios with hard energy budgets such as long-duration covert operations and battery-limited unmanned aerial relay platforms.

## Figures and Tables

**Table 1:** Channel model applicability for VHF operational environments.

Environment	Propagation Condition	Recommended Model	K-Factor Range	Reference
Maritime (open sea)	Strong LOS + Sea surface reflection	Rician/two-ray	6–12 dB	[33,34]
Aeronautical (air/ground)	Dominant LOS, limited scattering	Rician	10–20 dB	[35]
Urban mobile	Dense scattering, frequent NLOS	Rayleigh	K ≈ 0	[36]
Suburban/rural	Partial LOS, moderate multipath	Rician (low K)	3–8 dB	[37]
Mountainous/valley tactical	Terrain obstruction, NLOS dominant	Rayleigh	K ≈ 0	[36,37]

**Table 2:** Waveform configuration parameters.

Waveform	Channel Coding	Code Rate	Modulation	Interleaving
VHF_1	BCH(31,16)	0.516	BPSK	Matrix 31 × 31
VHF_2	RS(31,15)	0.484	32-FSK	Matrix 31 × 31
VHF_3	Golay(24,12)	0.500	BPSK	Matrix 24 × 24
VHF_4	RS(15,7)	0.467	16-QAM	Matrix 15 × 15
VHF_5	Turbo (R = 1/3)	0.333	BPSK	Random
VHF_6	Turbo (R = 1/3)	0.333	16-QAM	Random
VHF_7	RS(31,15)	0.484	BPSK	Matrix 31 × 31

Note: For VHF_5, the random interleaver uses a pseudo-random permutation of length N = 1024 bits, generated using a linear congruential generator with a fixed seed for reproducibility. VHF_6 and VHF_7 are included as comparative configurations to validate the design rationale of the core set. All seven waveforms are available as candidates in the 63-configuration optimization space (Section 4.6.1).

**Table 3:** BER values at representative SNR points under Rayleigh fading.

SNR (dB)	VHF_1 BCH + BPSK	VHF_2 RS + 32FSK	VHF_3 Golay + BFSK	VHF_4 RS + 16QAM	VHF_5 Turbo + BPSK
−5	0.319	0.375	0.245	0.204	0.348
−3	0.271	0.292	0.242	0.193	0.302
0	0.169	0.156	0.225	0.175	0.208
3	0.115	0.021	0.192	0.146	0.097
5	0.058	<10^−6^	0.178	0.120	0.030
7	0.033	<10^−6^	0.134	0.078	4.2 × 10^−3^
10	8.2 × 10^−3^	<10^−6^	0.059	0.025	1.0 × 10^−5^
15	1.7 × 10^−4^	<10^−6^	5.8 × 10^−3^	1.1 × 10^−3^	<10^−6^

**Table 4:** Simulation validation: BCH(31,16) + BPSK theoretical BER upper bound versus simulated BER under Rayleigh fading.

$E_{b}/N_{0}$ (dB)	Theoretical Upper Bound	Simulated BER	Relationship
5	6.08 × 10^−3^	3.50 × 10^−3^	Sim < Bound
7	1.08 × 10^−4^	6.45 × 10^−5^	Sim < Bound
10	1.45 × 10^−9^	<10^−6^ (floor)	Consistent

**Table 5:** Waveform simulation performance benchmark.

Waveform	Coding	Modulation	Decoding Algorithm	Simulation Speed	Real-Time Ratio
VHF_1	BCH(31,16)	BPSK	Berlekamp- Massey	~0.95 × real-time	≈1:1
VHF_2	RS(31,15)	32FSK	Berlekamp- Massey	~0.90 × real-time	≈1:1
VHF_3	Golay(24,12)	BFSK	Lookup table	~0.98 × real-time	≈1:1
VHF_4	RS(15,7)	16QAM	Berlekamp- Massey	~0.92 × real-time	≈1:1
VHF_5	Turbo (R = 1/3)	BPSK	Iterative MAP, 12 iter.	~0.10 × real-time	≈1:10

Note: Speed measured as ratio of simulated communication duration to wall-clock time for 10^6^ transmitted bits [43].

**Table 6:** SITL synchronization test parameters.

Parameter	Value
Test duration	24 h
Ping interval	1 s
Packet size	64 bytes
Physical device IP	192.168.43.211
Virtual node IP	192.168.43.200
Subnet mask	255.255.255.0
Packet filter	arp or icmp

**Table 7:** Aggregated pairwise comparison matrix.

	Voice Quality (VQ)	BER (BER)	Delay (CD)	Range (CR)
Voice Quality (VQ)	1	1.5	1.5	3
BER (BER)	0.67	1	1	2
Delay (CD)	0.67	1	1	2
Range (CR)	0.33	0.5	0.5	1

**Table 8:** Per-cycle computational complexity and timing of DT-MAB framework.

Component	Complexity	Per-Cycle Time	Memory
Lin-UCB UCB computation	$O\left(K\cdot d^{3}\right)$	<1 ms	10 KB ($A_{k}$, $b_{k}$ matrices)
Digital twin virtual exploration	1 simulation call	≈100 ms	depends on waveform (Table 5)
SITL synchronization	—	30 ms (mean)	<1 KB (state vector)
Total	—	<105 ms	<12 KB
Decision interval	—	1000 ms	—
Headroom	—	>9×	—

Notes: K = 63 (action space size), d = 4 (context dimension). Lin-UCB timing measured on Intel Core i7-10700 @ 2.9 GHz (Section 4.1). Digital twin simulation time dominated by Turbo MAP decoder (Table 5).

**Table 9:** Experimental scenario parameters (communication link and interference source).

Category	Parameter	Value
Communication Link	Link distance	5 km
	Operating frequency	30.525 MHz (λ ≈ 9.8 m)
	Transmitter power	5 W (37 dBm)
	Antenna type	Omnidirectional whip
	Antenna gain	3 dBi
	Receiver sensitivity	−100 dBm
	Receiver buffer	256 kbit
	Transmission rate	50 packets/s
Interference Source	Jammer center frequency	30 MHz
	Jammer bandwidth	1 kHz
	Interference type	Narrowband continuous wave (CW)
	Jammer power range	30–300 W
	Jammer mobility	Mobile, linear trajectory (see below)

**Table 10:** Reception performance versus interference power (mobile jammer, linear trajectory, 50 packets/s transmission rate).

Jammer Power (W)	Avg. Reception Rate (pkt/s)	Packet Loss Rate (%)	Effective Link Status
0 (baseline)	48.5	3	Fully operational
30	25	50	Degraded but functional
50	20	60	Severely degraded
80	8	84	Near denial
300	2	96	Effectively denied

**Table 11:** Phase-averaged reception rate (pkt/s) across six methods under three-phase experimental scenario.

Method	Phase 1 (No Jamming)	Phase 2 (30 W Jamming)	Phase 3 (Recovery)
Oracle	29.2 ± 0.1	18.0 ± 0.1	29.2 ± 0.1
DT-MAB	17.0 ± 7.2	8.7 ± 1.8	16.1 ± 3.2
MAB-only	11.2 ± 0.3	4.5 ± 0.2	12.8 ± 0.8
DT-PSO	13.6 ± 0.3	7.5 ± 0.6	13.9 ± 0.8
Rule-Based	8.0 ± 0.2	1.5 ± 0.2	8.8 ± 0.2
Fixed	3.8 ± 0.1	0.0 ± 0.0	4.5 ± 0.1

Note: The high variance of DT-MAB (e.g., ±7.2 in Phase 1) reflects exploration-dependent learning trajectories inherent to contextual bandits in large action spaces; the mean performance consistently exceeds all baselines.

**Table 12:** Ablation experiment: DT-MAB versus MAB-only (without digital twin virtual exploration).

Metric	DT-MAB	MAB-Only	Improvement
Phase 1 avg. reception rate (pkt/s)	17.0 ± 7.2	11.2 ± 0.3	+52%
Phase 3 avg. reception rate (pkt/s)	16.1 ± 3.2	12.8 ± 0.8	+26%
Performance drop events (<15 pkt/s)	250 ± 79	373 ± 6	−33%
Cumulative regret (500 rounds)	5645 ± 1621	7951 ± 112	−29%

**Table 13:** Mean transmit power, reception rate, and energy efficiency across six methods (mean ± std over 10 random seeds, 500 rounds).

Method	Mean Tx Power (W)	Reception Rate (pkt/s)	Energy Efficiency (pkt/(s·W))
Oracle (upper bound)	14.30 ± 0.19	24.7 ± 0.1	1.731 ± 0.025
DT-MAB (proposed)	14.36 ± 3.01	13.5 ± 3.2	0.933 ± 0.081
DT-PSO	15.02 ± 0.18	11.2 ± 0.3	0.747 ± 0.019
MAB-only	13.01 ± 0.18	8.8 ± 0.2	0.680 ± 0.019
Rule-Based	6.29 ± 0.05	5.5 ± 0.1	0.880 ± 0.017
Fixed (10 W)	10.00 ± 0.00	2.4 ± 0.0	0.243 ± 0.003

Notes: Energy efficiency = (mean reception rate)/(mean transmit power). Higher values indicate more packets received per unit of transmit energy. DT-MAB’s high standard deviation in mean Tx power (±3.01 W) reflects the exploration-dependent learning trajectory across seeds, contrasting with the deterministic per-cycle optimization of DT-PSO and Oracle.

## Data Availability

The data presented in this study are available in the article. The study did not generate new publicly available datasets.
